# Caesarean section surgical techniques: 3 year follow-up of the CORONIS fractional, factorial, unmasked, randomised controlled trial

**DOI:** 10.1016/S0140-6736(16)00204-X

**Published:** 2016-07-02

**Authors:** The CORONIS collaborative group

## Abstract

**Background:**

The CORONIS trial reported differences in short-term maternal morbidity when comparing five pairs of alternative surgical techniques for caesarean section. Here we report outcomes at 3 years follow-up.

**Methods:**

The CORONIS trial was a pragmatic international 2 × 2 × 2 × 2× 2 non-regular fractional, factorial, unmasked, randomised controlled trial done at 19 sites in Argentina, Chile, Ghana, India, Kenya, Pakistan, and Sudan. Pregnant women were eligible if they were to undergo their first or second caesarean section through a planned transverse abdominal incision. Women were randomly assigned by a secure web-based allocation system to one intervention from each of the three assigned pairs. All investigators, surgeons, and participants were unmasked to treatment allocation. In this follow-up study, we compared outcomes at 3 years following blunt versus sharp abdominal entry, exteriorisation of the uterus for repair versus intra-abdominal repair, single versus double layer closure of the uterus, closure versus non-closure of the peritoneum, and chromic catgut versus polyglactin-910 for uterine repair. Outcomes included pelvic pain; deep dyspareunia; hysterectomy and outcomes of subsequent pregnancies. Outcomes were assessed masked to the original trial allocation. This trial is registered with the Current Controlled Trials registry, number ISRCTN31089967.

**Findings:**

Between Sept 1, 2011, and Sept 30, 2014, 13 153 (84%) women were followed-up for a mean duration of 3·8 years (SD 0·86). For blunt versus sharp abdominal entry there was no evidence of a difference in risk of abdominal hernias (adjusted RR 0·66; 95% CI 0·39–1·11). We also recorded no evidence of a difference in risk of death or serious morbidity of the children born at the time of trial entry (0·99, 0·83–1·17). For exteriorisation of the uterus versus intra-abdominal repair there was no evidence of a difference in risk of infertility (0·91, 0·71–1·18) or of ectopic pregnancy (0·50, 0·15–1·66). For single versus double layer closure of the uterus there was no evidence of a difference in maternal death (0·78, 0·46–1·32) or a composite of pregnancy complications (1·20, 0·75–1·90). For closure versus non-closure of the peritoneum there was no evidence of a difference in any outcomes relating to symptoms associated with pelvic adhesions such as infertility (0·80, 0·61–1·06). For chromic catgut versus polyglactin-910 sutures there was no evidence of a difference in the main comparisons for adverse pregnancy outcomes in a subsequent pregnancy, such as uterine rupture (3·05, 0·32–29·29). Overall, severe adverse outcomes were uncommon in these settings.

**Interpretation:**

Although our study was not powered to detect modest differences in rare but serious events, there was no evidence to favour one technique over another. Other considerations will probably affect clinical practice, such as the time and cost saving of different approaches.

**Funding:**

UK Medical Research Council and the Department for International Development.

## Introduction

Caesarean section is one of the most commonly undertaken operations worldwide and is not done in a standardised way. In the CORONIS trial, we previously reported the short-term outcomes associated with different surgical techniques at caesarean section in 15 935 women in low-income and middle-income settings.^1^ We compared blunt versus sharp abdominal entry, exteriorisation of the uterus for repair versus intra-abdominal repair, single versus double layer closure of the uterus, closure versus non-closure of the peritoneum (pelvic and parietal), and chromic catgut versus polyglactin-910 for uterine repair. Our findings showed no clear benefits of any of the randomised comparisons on a range of short-term outcomes (up to 6 weeks after the surgery). Many of the important maternal outcomes associated with different surgical techniques will be apparent in the longer term, including the functional integrity of the uterine and abdominal scar during subsequent pregnancies and other long-term postoperative effects such as chronic pelvic pain, infertility, and symptoms related to peritoneal and bowel adhesions, including bowel obstruction.

In this CORONIS follow-up study, we aimed to measure and compare the incidence of outcomes between the groups of women who took part in the CORONIS trial at least 3 years after their CORONIS caesarean section. The protocol for the follow-up study has been published.^2^

**Research in context**

**Evidence before this study**

Before this follow-up study, several reviews, including Cochrane systematic reviews, had synthesised available evidence of the different surgical techniques included in the CORONIS trial. Based on this evidence, clinical practice guidelines had been made with recommendations about the different approaches. However, most evidence for this guidance came from studies that rarely measured outcomes beyond the immediate postoperative period. With regards to blunt versus sharp abdominal entry, for exteriorisation versus intra-abdominal repair of the uterus, no studies compared long-term outcomes for this intervention pair. For single versus double layer closure of the uterine incision, uterine scar integrity after single versus double layer uterine closure had been assessed in the longer term. A systematic review of observational studies failed to find evidence of a clear association between single layer closure and uterine rupture. For closure versus non-closure of the peritoneum and for subsequent intra-abdominal adhesions, studies only followed up women who had a subsequent laparotomy (including a repeat caesarean section) or laparoscopy. Two systematic reviews looked at the longer term effect of closure versus non-closure of the peritoneum on adhesion formation. Both reviews agreed that the available evidence is limited because most findings came from small observational studies that had a high risk of bias; the investigators suggested in their conclusions that non-closure of the peritoneum was associated with an increased risk of intra-abdominal adhesions, of all grades. For chromic catgut versus polyglactin-910 for uterine repair, no studies compared long term outcomes for this intervention pair.

**Added value of this study**

Our results provide the first evidence of the long-term effects of exteriorisation versus intra-abdominal repair of the uterus, and for chromic catgut versus polyglactin-910 for repair of the uterus. For sharp versus blunt abdominal entry, the risk of incisional hernias after caesarean section has been studied in the Danish population. There were 134 hernias recorded in 57 564 women (a 10 year incidence of 0·2%). The study was not able to establish whether the abdominal entry approach used at the time of the caesarean section was blunt or sharp. Therefore, CORONIS provides evidence that the risk of later hernia formation is very small, and we noted no difference in the risk between the two approaches. For closure versus non-closure of the peritoneum, the outcome measures in previous studies vary, but rely on finding evidence of pelvic adhesions. The relevant Cochrane review includes follow-up studies from four randomised trials that find no evidence of an increased risk of adhesions, although the studies included are small. Because intra-abdominal adhesions are often asymptomatic, it is difficult to interpret much of this evidence. In the CORONIS study we noted no difference in symptoms reported by more than 8000 women assessed in this intervention pair, suggesting that even if there are differences in the incidence of adhesion formation, these are unlikely to produce symptomatic disease for most women.

**Implications of all the available evidence**

Polyglactin-910 costs at least twice as much as chromic catgut and the finding of no benefit with the use of polyglactin-910 suggests that chromic catgut should be the suture material of choice for uterine repair in appropriate settings. The absence of evidence of any difference between closure versus non-closure of the peritoneum also suggests that non-closure is to be preferred, not simply because it may save time as previous recommendations suggest, but because it will result in the use of fewer sutures, therefore decreasing costs. The use of routine exteriorisation to repair the uterus can also be discouraged as it confers no benefit. If double layer closure of the uterus frequently employs two sutures, then single layer closure would also decrease costs. For those surgeons who routinely repair the uterus in two layers with a single suture, the only potential impact on the operation is the time taken, although there were no apparent differences in the duration of the operation between single and double layer closure in CORONIS.

Other implications include that the techniques used at caesarean section do not need to be modified by whether the caesarean section is done before or after the onset of labour, or by whether the caesarean section being performed is the first or second caesarean section.

## Methods

### Study design and participants

The methods of the trial have already been described, but in summary, women were eligible for this 2 × 2 × 2 × 2 × 2 non-regular fractional factorial randomised trial if they were undergoing delivery by lower segment caesarean section through a transverse abdominal incision, and had no more than one previous caesarean section, and there was no clear indication for a particular surgical technique or material to be used.^2^ Women were not eligible for the follow-up study if they had been randomly assigned in error, withdrawn consent from the trial, delivered vaginally at the time of recruitment to CORONIS, opted out of the follow-up study, no data were collected after trial entry.

The study was approved by Oxford Tropical Research Ethics Committee (OXTREC; 013-06a) and by the relevant research ethics committees in each of the participating countries and sites. The CORONIS follow-up study steering committee provided independent oversight.

### Data collection

Telephone contact was maintained regularly with all women eligible for follow-up throughout the follow-up period to facilitate a face-to-face assessment at least 3 years after their CORONIS caesarean section. During the assessment, a detailed medical history was taken from the woman by a specifically trained health professional. Details of the woman's general and reproductive health and history of all subsequent pregnancies were recorded on the health assessment questionnaire.^3^ Any hospital admissions for a specified range of disorders (eg, subsequent pregnancy complications, including uterine rupture or dehiscence, hysterectomy, placenta praevia, or other morbidity including non-pregnancy related hysterectomy, and laparoscopy or laparotomy) were followed up by a review of the hospital notes and completion of the relevant event report form, wherever possible.^4^ Not all women could be seen in person, and telephone follow-up interviews were undertaken when a face-to-face assessment was not possible.

### Outcomes

The outcomes measured for the follow-up study were: following the CORONIS birth and before any subsequent pregnancy: any new onset or worsening of pelvic pain; dysmenorrhoea; deep dyspareunia; urinary symptoms; diagnostic laparoscopy or diagnostic laparotomy (not related to pregnancy); hysterectomy or tubal or ovarian surgery (not related to pregnancy); bladder or bowel damage in those women who had surgery, excluding diagnostic laparoscopy and diagnostic laparotomy (not related to pregnancy); after the CORONIS birth and before any subsequent pregnancy, any new onset of abdominal hernia; bowel obstruction; woman's death; number of women with no subsequent pregnancy; voluntary infertility; involuntary infertility; use of fertility treatments; number of women having any subsequent pregnancy and for these women, the following outcomes were measured: inter-pregnancy interval from the CORONIS birth to the end of the subsequent pregnancy (regardless of loss or birth); miscarriage of the pregnancy subsequent to the CORONIS birth; ectopic pregnancy; for the birth following the CORONIS birth: gestation at delivery (by best estimate) of the first viable pregnancy (viable pregnancy defined as gestational age > 24 or >28 weeks depending on country specific definition); stillbirth; neonatal death; mode of delivery as non-instrumental vaginal; instrumental vaginal; pre-labour caesarean section; in labour caesarean section; other pregnancy complications including: uterine rupture, uterine scar dehiscence, placenta praevia, morbidly adherent placenta, abruption, postpartum haemorrhage requiring transfusion, severe infection within 6 weeks post partum, hysterectomy up to 6 weeks post partum, manual removal of placenta; bladder or bowel damage at the time of subsequent caesarean section; death or serious morbidity of the child who was born at the time of CORONIS participation (for the sharp *vs* blunt abdominal entry intervention pair).

Many outcomes are potentially relevant for each intervention pair; however, some outcomes are more likely to be affected by some interventions than others. For example, interventions such as closure or non-closure of the peritoneum can have an effect on subsequent pelvic adhesion formation, so outcomes such as infertility and pelvic pain were regarded as most important for this intervention pair. For single versus double layer closure of the uterus, the main outcomes of interest relate to subsequent pregnancy, whereas outcomes such as dyspareunia and infertility were deemed of secondary interest. Therefore, we categorised comparisons into main comparisons of interest and secondary comparisons of interest. This approach was pre-specified considering biological plausibility and to account for the dangers of multiple testing. The main comparisons of interest are reported here and the remaining comparisons, along with a description of the process for agreeing the main and secondary comparisons of interest, are in the appendix.

### Statistical analysis

The original trial recruited 15 935 women, with at least 9000 women in each intervention pair. We assumed an overall response rate of at least 80% for the follow-up, and an assumption that 80% of these women would have a subsequent pregnancy during the follow-up, which would result in nearly 6000 women in each intervention pair with a subsequent pregnancy. This number would be sufficient to detect modest but clinically important differences between any principal comparisons for this population. The statistical power available, based on a fixed sample size, for a range of event rates on a selection of outcomes was high (>90%), although there were no published data for estimates for many of the outcomes of interest from low-resource and middle-resource settings.^2^, ^5^, ^6^

A detailed statistical analysis plan was developed and approved by the study steering committee before the final analysis. All outcomes were analysed in the groups into which the women were randomly allocated irrespective of the technique received.

Baseline demographic and clinical characteristics are described separately for the five intervention pairs. Outcome variables were derived with information collected on the Health Assessment Questionnaire. Because clinical details of hospital admissions would not be known by most participants during the medical history interview, data for the event report form were deemed more reliable, when available. All comparative analyses were done adjusting for the minimisation factors in-labour or not in-labour caesarean section and number of previous caesarean sections (none or one) where possible. Binary outcomes were analysed with log binomial regression models and results presented as adjusted risk ratios. Continuous outcomes were analysed with linear regression models and results presented as adjusted differences in means. Time to the first subsequent pregnancy after CORONIS used Cox regression methods and a hazard ratio is presented. For the main comparisons, 95% CIs are given and 99% CIs for secondary comparisons.

To facilitate the interpretation of the findings appropriate to the outcome analysed, the analysis populations varied and were prespecified in the statistical analysis plan. Outcomes relating to deaths of women or children in CORONIS were based on all women eligible for the follow-up study (ie, women eligible for assessment). For most of the remaining outcomes, analyses were based on all surviving women for whom follow-up data were available (women assessed). Pregnancy related outcomes for which viability was not relevant (eg, ectopic pregnancy) were based on women with a subsequent pregnancy. To take account of the potential for differences in the pregnancy rate between any two interventions being compared, we analysed the outcomes relating to a subsequent viable pregnancy, such as uterine rupture, with two populations: women who subsequently had at least one viable pregnancy as a primary analysis and women assessed as a secondary analysis.

The primary analysis of the outcome death or serious morbidity of the CORONIS children excluded stillbirths. We also did a secondary analysis including stillbirths. Outcomes for infants from multiple births were expected to be correlated and therefore a clustering effect might be anticipated. For the primary analysis of all neonatal and child outcomes, all babies or CORONIS children were treated as independent.^7^ A sensitivity analysis assessed the effect of allowing for potential clustering.

Prespecified subgroup analyses were in-labour or not in-labour caesarean section and number of previous caesarean sections (none or one). Stratum-specific effect estimates with 95% CIs are presented on forest plots, by pair of interventions, with a test for interaction. Plausible treatment interactions were difficult to pre-specify. The same strategy for the analysis of interactions for the short term outcomes was employed for this report of the long term outcomes—ie, analyses of interactions would only be done for the main comparisons; and three-way interactions would not be investigated unless there was strong evidence of a two-way interaction in the presence of main effects.

Sensitivity analyses on the main comparisons of interest were pre-specified relating to the timing of the completion of the follow-up assessment: first, analyses were restricted to a follow-up window of 3 years (within 3 months); to address variations in time at risk, we used Cox regression methods for outcomes related to delivery using date of randomisation and date of delivery to indicate the time at risk; to investigate the consistency of effect over time to follow-up for outcomes not related to delivery, time to follow-up was dichotomised with cutoff points driven by the data, and subgroup analyses were done using tests of interaction.

Generalisability of each of the analysis populations compared with those not included was assessed on selected characteristics of the women at trial entry and short-term outcomes using χ^2^ tests, overall, and by intervention pair.

All analyses were done with Stata/SE (version 13). The CORONIS trial is registered with Current Controlled Trials, number ISRCTN31089967.

### Role of the funding source

The funders of the study had no role in the design, data collection, analysis, interpretation, or writing of the report. The corresponding author had full access to all the data in the study and had final responsibility for the decision to submit for publication.

## Results

**Figure 1:**
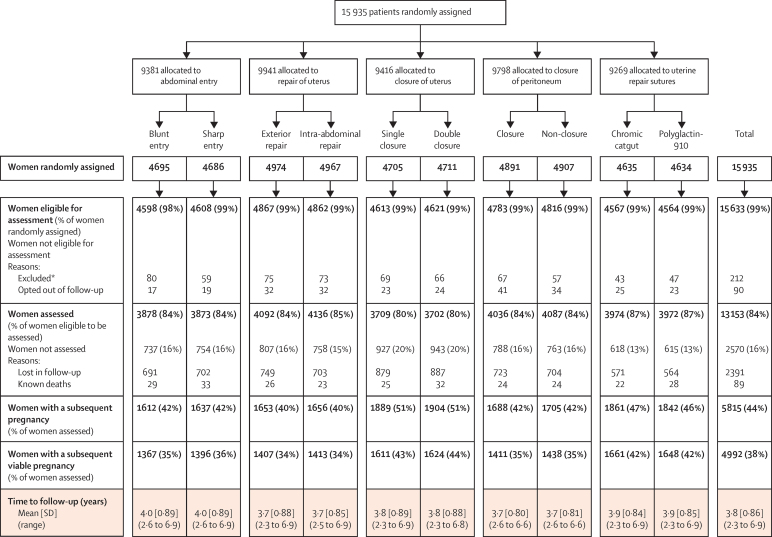
Participant flow diagram *Reasons for exclusion: women randomly assigned in error, women who withdrew consent from main trial, baseline data not received, vaginal deliveries at the time of recruitment to CORONIS. Six additional cases of women randomly assigned in error have been found since publication of the main results.

Between Sept 1, 2011, and Sept 30, 2014, 13 153 (84%) of 15 633 women were followed up in Argentina, Chile, Ghana, India, Kenya, Pakistan, and Sudan (figure 1, appendix). Of the women eligible for follow-up, very few women opted out (90/15 633 [1%]), and 2391 (15%) could not be assessed. 89 women were known to have died from the time of trial entry to the time of follow-up (figure 1, appendix).

The mean duration between trial entry and follow-up was 3·8 years (SD 0·86; range 2·3–6·9; figure 1). 11 326 (86%) women had a formal face-to-face assessment and 1827 (14%) were interviewed by telephone. Of 380 women who had a hospital admission for a specified range of disorders, hospital notes could be checked for 328 (86%) of them. 5815 (44%) of 13 153 women had a subsequent pregnancy during the period of follow-up, which was substantially lower than anticipated. Of the 4992 women who had a subsequent viable pregnancy, 3328 (66%) had a repeat caesarean section before labour starting.

The characteristics and selected short-term outcomes of women assessed in the study were well balanced between the randomised groups of the study (appendix). Those women assessed in the follow-up study were broadly similar to those not included. However, women included were slightly older, were more likely to have had their second caesarean section at the time of trial entry, were more likely to have had their CORONIS caesarean section during labour, and were less likely to have experienced a stillbirth or early death of their CORONIS baby. Importantly, there was no difference in the incidence of the primary outcome between those included and those not included in the analysis (appendix).

The outcomes reported for different intervention pairs varied based on a prespecified assessment of biological plausibility. Table 1, Table 2, Table 3, Table 4, Table 5 show the primary analysis of the main comparisons of interest. For blunt versus sharp abdominal entry, there was no evidence of a difference in the risk of abdominal hernias in women and no evidence of a difference in the risk of death or serious morbidity in the children born at the time of trial entry (table 1). This latter finding was unchanged in the secondary analysis, which included stillbirths with death or serious morbidity (appendix). For exteriorisation of the uterus versus intra-abdominal repair, we noted no evidence of a difference in the risk of involuntary infertility or ectopic pregnancy (table 2). For single versus double layer closure of the uterus, there was no evidence of a difference in maternal death, or a range of adverse pregnancy outcomes including uterine rupture or dehiscence. This finding was unchanged when the denominator was all women assessed (appendix). The absolute risk of uterine rupture was very low (one per 1000 women with a subsequent pregnancy), as was the risk for many other serious adverse outcomes in a subsequent viable pregnancy (table 3). For closure versus non-closure of the peritoneum, outcomes relating to symptoms associated with pelvic adhesions did not differ between groups (table 4). For chromic catgut versus polyglactin-910 sutures for uterine repair, we noted no evidence of a difference in the main comparisons for adverse pregnancy outcomes in a subsequent pregnancy (table 5). This finding was unchanged when the denominator was all women assessed (appendix). Similarly none of the secondary comparisons for all the intervention pairs differed between groups (appendix).

**Figure 2:**
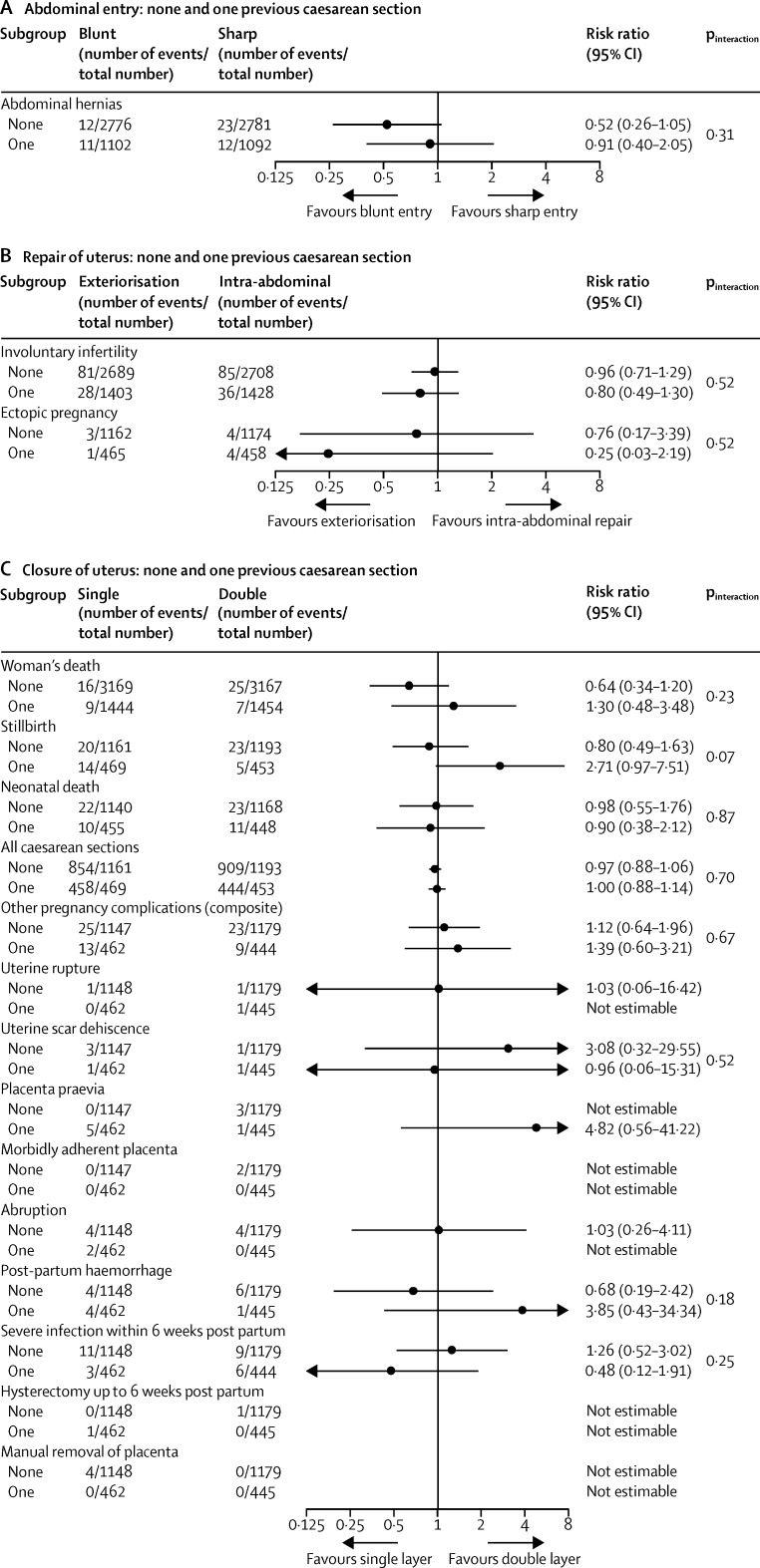
Subgroup analyses of women with no or one previous caesarean section for (A) abdominal entry (blunt *vs* sharp), (B) repair of uterus (exteriorisation *vs* intra-abdominal), and (C) closure of uterus (single *vs* double layer)

**Figure 3:**
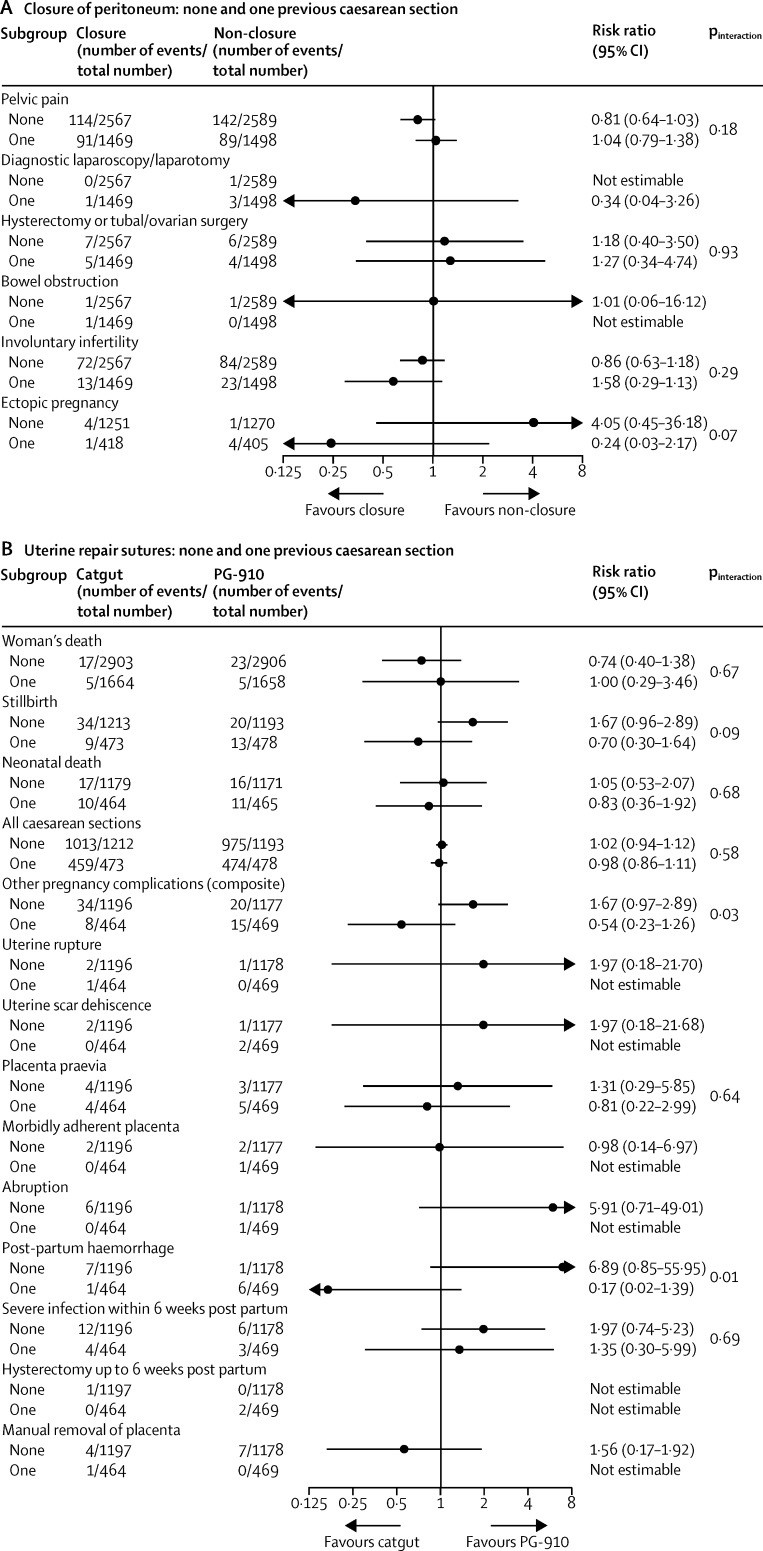
Subgroup analyses of women with no or one previous caesarean section for (A) closure of peritoneum (closure *vs* non-closure), (B) uterine repair sutures (catgut *vs* PG-910)

**Figure 4:**
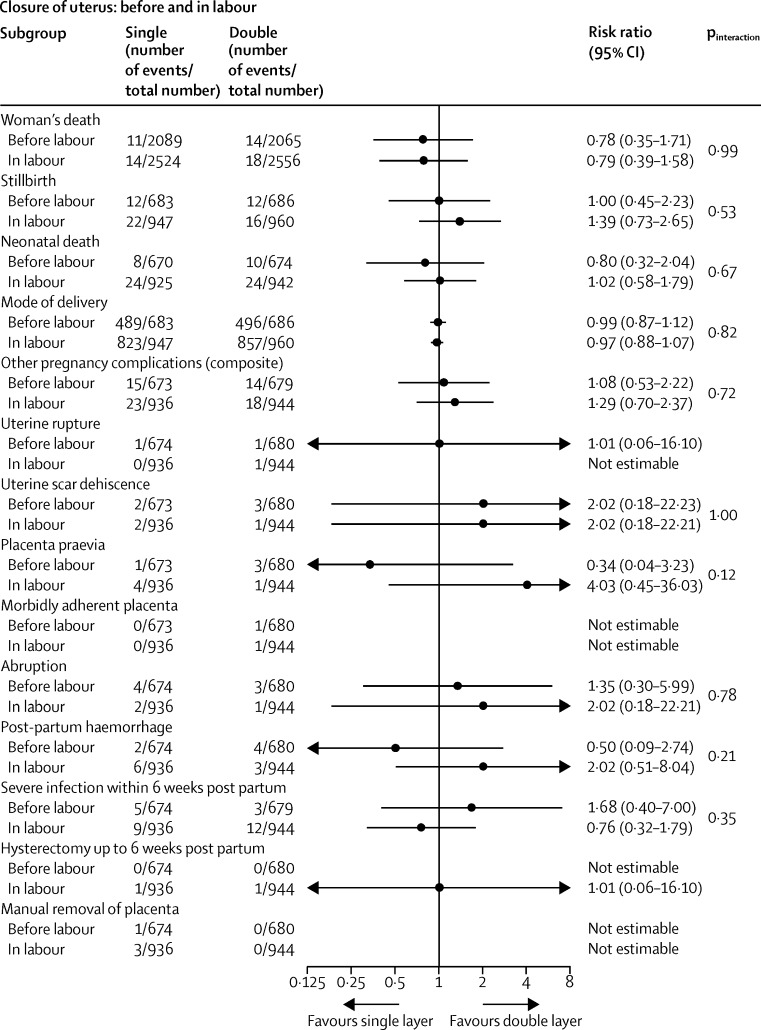
Subgroup analyses of women with caesarean section before or in labour for closure of uterus (single *vs* double layer)

**Figure 5:**
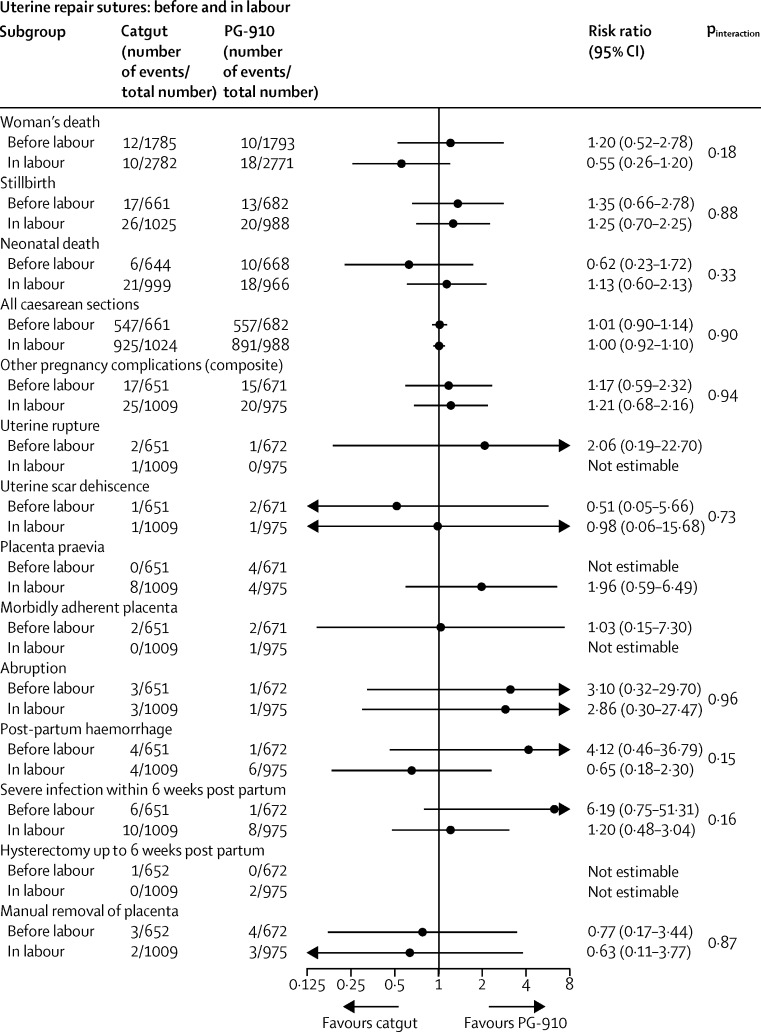
Subgroup analyses of women with caesarean section before or in labour for uterine repair sutures (catgut *vs* PG-910)

The prespecified subgroup analyses showed similar effects across the strata used (Figure 2, Figure 3, Figure 4, Figure 5). The one exception was chromic catgut versus polyglactin-910 for uterine repair by none versus one caesarean section on the composite outcome of other pregnancy outcomes (test for interaction p=0·03), and one of its components, postpartum haemorrhage (test for interaction p=0·01; figure 3B). This finding suggests there might be a differential effect, with polyglactin-910 favoured for the first caesarean section and chromic catgut favoured for the second caesarean section. In view of the fact that we recorded no effect for this outcome overall and that the number of events is very small, this significant difference might be spurious.

Sensitivity analyses on all the main comparisons noted similar effect sizes and confidence intervals (not presented, available on request). In view of the perception that substantial risks are associated with caesarean section, especially during subsequent pregnancies, and the paucity of data for these risks in many of these countries, it was surprising to note how uncommon severe adverse outcomes were, although the data suggest some regional variation in the incidence of these disorders (appendix). In particular, we recorded a low overall incidence of problems with placentation, such as morbidly adherent placenta, or placenta praevia.

## Discussion

The CORONIS trial compared five intervention pairs that were deemed the most important unanswered questions about the surgical techniques used at caesarean section in low-income and middle-income country settings. Despite the very large size of this trial, we did not find one technique that results in improved outcomes for women when compared with the others.

The main strengths of this study are its size, the completeness of follow-up, the rigorous data collection process, and the participation of several countries. CORONIS is the largest trial undertaken so far to investigate the effect of using different surgical techniques for caesarean section, and having 84% of eligible women followed up by a detailed medical interview at least 3 years after their caesarean section, with checking of hospital admission notes, represents a very substantial undertaking. The trial was done in Africa, South America and south Asia, which gives the results broad generalisability across the different country settings.

However, our study has several limitations. The participating centres were generally large referral hospitals with an active interest in research. The incidence of adverse outcomes might be higher in other settings, such as in less well-resourced hospitals. However, for several outcomes, incidence varied between different settings (appendix); however, the absence of any detectable effect was consistent across different settings, suggesting that the results will be generalisable given a reasonable range of incidence rates.

The incidence of a subsequent pregnancy in this group of women was lower than originally anticipated. Although there were no data to inform the likely subsequent pregnancy rate in the participating countries, we estimated that up to 80% of women would have a pregnancy in the 3 years after trial entry. The incidence of pregnancy was 44%, which limited the power of the study to detect differences in outcomes between the intervention pairs. Additionally, the incidence of caesarean section before labour onset in a subsequent pregnancy was very high. As might be expected, almost all women recruited to the trial at their second caesarean section went on to deliver by caesarean section for their subsequent births. However, many women who had their first caesarean section at the time of recruitment to the trial also went on to have a pre-labour caesarean section in their subsequent pregnancy. This outcome probably accounted for the very low incidence of several adverse pregnancy outcomes such as uterine rupture, which is strongly associated with labour.^8^ Similarly, a long interpregnancy interval is protective against uterine rupture and women in this study had a longer interval between pregnancies than anticipated, perhaps because these sites gave clear advice to avoid pregnancy for at least 1 year (and in some instances 2 years) after a caesarean section. The incidence of non-pregnancy related outcomes was also lower than anticipated. This finding limits the power of the study to detect anything but large effect sizes, but does provide reassurance that the risk of outcomes after caesarean section in these settings is generally low.

Despite the high quality of care provided in the participating sites, medical note taking and archiving were not always thorough. Notes were found for 86% of the 380 women when these were sought, but often they contained little detail at subsequent surgery, especially in relation to the presence or absence of pelvic adhesions, which was only mentioned in 41 sets of notes. Similarly, recording of the number of units given during blood transfusions was often poor. This was complicated further by women presenting with later morbidity or subsequent pregnancies at hospitals other than those where they had been recruited.

However, despite these limitations, this study provides important insights into the epidemiology of outcomes after caesarean section within these different settings. Our findings provide a clearer understanding of later morbidity, especially in relation to subsequent births, and for the first time, allows quantification of the risk of a range of long term outcomes associated with caesarean section.

We noted some benefit associated with chromic catgut when compared with polyglactin-910 for uterine repair in the immediate post-caesarean section period in our previous report.^1^ The finding of no difference between suture materials in the longer term is less surprising. The short-term difference recorded was an increased risk of blood transfusion in the polyglactin-910 group. This increase would be unlikely to result in longer term effects unless increased short-term bleeding at the uterine incision site weakened the integrity of the uterine scar for the next pregnancy. The finding of a significant difference in the incidence of postpartum haemorrhage in the subgroup analysis with polyglactin-910 being favoured for the first caesarean section and chromic catgut being favoured for the second caesarean section has little biological plausibility and might be a spurious finding. However, this association between suture material and haemorrhage complications in both short term and long term outcome is intriguing.

The publication of the short-term outcomes of this trial showed the importance of considering the potentially more important longer term consequences of different caesarean section techniques before making recommendations for clinical practice. Despite the lack of power to detect modest differences in rare, but serous events, there is no evidence to favour one technique over another with respect to a range of clinical outcomes. Therefore, other considerations will probably guide clinical practice, such as surgeon preference and economic and organisation factors. Furthermore, the implications for clinical practice are becoming clearer. 9, 10, 11 For example, with respect to the suture material used for uterine scar closure, there is no evidence to support the use of the substantially more expensive polyglactin-910. Additionally, current recommendations that state that blunt abdominal entry should be preferred over sharp entry are not supported by this, the largest trial ever undertaken addressing this comparison.^12^, ^13^ Although some studies have shown that blunt abdominal entry can also result in a shorter operating time, this does not seem to lead to measurable improvements in clinical outcomes. However, shorter operating times might lead to more effective use of theatre time in centres with large numbers of caesarean sections being done. Additionally, guidance stating that peritoneal closure is not recommended cannot be supported on the grounds of the clinical superiority of non-closure.^14^, ^15^, ^16^ The use of less suture material could decrease costs, which might be a major consideration in resource-poor countries. Non-closure also results in a shorter operating time that could offer organisational advantages.

We were unable to detect any differences in the risk of uterine rupture or dehiscence when comparing single versus double layer uterine closure, which means that this debate will continue. It seems unlikely that any further trial of sufficient size will be undertaken to address this question, making us reliant on observational studies to explore this hypothesis.^17^, ^18^, ^19^, ^20^, ^21^, ^22^ In view of the level of detail available in routine notes in the settings in which this trial was undertaken, as well as in those of a similar trial in the UK,^23^ this will be a difficult question to answer. What we can be sure of is that like other serious consequences of caesarean section, uterine rupture is very rare, so the population effect of the different techniques used at caesarean section on subsequent adverse outcomes such as perinatal mortality will be very small.

During the original trial we collected details about all the surgical techniques used for the caesarean section. We now have the opportunity to explore what other aspects of the operation, other than those tested within CORONIS, might be worth exploring in subsequent trials in terms of their effect on both short-term and long-term outcomes for women.

Correspondence to: Prof Peter Brocklehurst, Institute for Women's Health, University College London, London WC1E 6AU, UK p.brocklehurst@ucl.ac.uk

## Figures and Tables

**Table 1:** Outcomes associated with abdominal entry

	**Blunt abdominal entry**	**Sharp abdominal entry**	**Adjusted risk ratio (95% CI)**
**Women's health**			
Women assessed	3878	3873	..
Abdominal hernias*	23/3878 (<1%)	35/3873 (<1%)	0·66 (0·39–1·11)
**CORONIS children**			
Liveborn children of women eligible to be assessed	4725	4772	..
Known deaths or serious morbidity of CORONIS children	244/4725 (5%)	249/4772 (5%)	0·99 (0·83–1·17)

* After the CORONIS birth, any new onset.

**Table 2:** Outcomes associated with repair of uterus

	**Exterior repair**	**Intra-abdominal repair**	**Adjusted risk ratio (95% CI)**
**Reproductive status**			
Women assessed	4092	4136	..
Involuntary infertility	109/4092 (3%)	121/4136 (3%)	0·91 (0·71–1·18)
Use of fertility treatments	9/4090 (<1%)	15/4136 (<1%)	0·61 (0·27–1·38)
**Subsequent pregnancies**			
Women with a subsequent pregnancy*	1627	1632	..
Ectopic pregnancy	4/1627 (<1%)	8/1632 (<1%)	0·50 (0·15–1·66)

* Excludes 26 women with exteriorisation and 24 women with intra-abdominal repair, who were pregnant at time of assessment. Missing data are <1% unless otherwise stated.

**Table 3:** Outcomes associated with closure of uterus

		**Single closure**	**Double closure**	**Adjusted risk ratio (95% CI)**
Women's health				
	Women eligible to be assessed	4613	4621	..
	Known deaths	25/4613 (<1%)	32/4621 (<1%)	0·78 (0·46–1·32)
Subsequent pregnancies				
	Babies of women with a subsequent viable pregnancy*	1630	1646	..
	Stillbirth†	34/1630 (2%)	28/1646 (2%)	1·23 (0·75–2·01)
	Neonatal death†‡	32/1595 (2%)	34/1616 (2%)	0·96 (0·59–1·54)
Method of delivery†				
	Non-instrumental vaginal	309/1630 (19%)	288/1646 (18%)	..
	Instrumental vaginal	9/1630 (<1%)	5/1646 (<1%)	..
	Pre-labour caesarean section	1025/1630 (63%)	1076/1646 (65%)	..
	In labour caesarean section	287/1630 (18%)	277/1646 (17%)	..
All caesarean sections		1312/1630 (81%)	1353/1646 (82%)	0·98 (0·95–1·01)
Women with a subsequent viable pregnancy*		1611	1624	..
Other pregnancy complications, composite†§		38/1609 (2%)	32/1623 (2%)	1·20 (0·75–1·90)
	Uterine rupture†	1/1610 (<1%)	2/1624 (<1%)	0·50 (0·05–5·51)
	Uterine scar dehiscence†	4/1609 (<1%)	2/1624 (<1%)	2·01 (0·37–10·95)
	Placenta praevia†	5/1609 (<1%)	4/1624 (<1%)	1·23 (0·33–4·57)
	Morbidly adherent placenta†	0/1609	2/1624 (<1%)	-
	Abruption†	6/1610 (<1%)	4/1624 (<1%)	1·51 (0·43–5·35)
	Post-partum haemorrhage requiring transfusion of >1 unit of whole blood or packed cells†	8/1610 (<1%)	7/1624 (<1%)	1·15 (0·42–3·16)
	Severe infection within 6 weeks post partum†	14/1610 (<1%)	15/1623 (<1%)	0·94 (0·46–1·94)
	Hysterectomy up to 6 weeks post partum†	1/1610 (<1%)	1/1624 (<1%)	1·00 (0·06–15·90)
	Manual removal of placenta†	4/1610 (<1%)	0/1624	..

Missing data are <1% unless otherwise stated.

* Viability could not be assessed if gestational age was not recorded.

† For the birth after the CORONIS birth.

‡ Excludes stillbirths.

§ Includes: uterine rupture, uterine scar dehiscence, placenta praevia, morbidly adherent placenta, abruption, post-partum haemorrhage requiring transfusion, severe infection within 6 weeks, post-partum hysterectomy up to 6 weeks post partum, manual removal of placenta.

**Table 4:** Outcomes associated with closure of peritoneum

		**Closure of peritoneum**	**Non-closure of peritoneum**	**Adjusted risk ratio (95% CI)**
**Women's health**				
Women assessed		4036	4087	..
	Pelvic pain*	205/4036 (5%)	231/4087 (6%)	0·90 (0·75–1·08)
	Deep dyspareunia*	145/4036 (4%)	134/4087 (3%)	1·09 (0·87–1·38)
	Diagnostic laparoscopy/laparotomy†	1/4036 (<1%)	4/4087 (<1%)	0·25 (0·03–2·26)
	Hysterectomy or tubal/ovarian surgery†	12/4036 (<1%)	10/4087 (<1%)	1·21 (0·53–2·81)
	Bladder or bowel damage following surgery†‡	0/100	0/117	..
	Bowel obstruction§	2/4036 (<1%)	1/4087 (<1%)	2·03 (0·18–22·38)
**Reproductive status**				
Women assessed		4036	4087	..
	Involuntary infertility	85/4036 (2%)	107/4087 (3%)	0·80 (0·61–1·06)
	Use of fertility treatments	10/4035 (<1%)	12/4086 (<1%)	0·84 (0·36–1·94)
**Subsequent pregnancies**				
Women with a subsequent pregnancy (excludes 17 closure, 30 non-closure women pregnant at time of assessment)		1669	1675	..
Ectopic pregnancy		5/1669 (<1%)	5/1675 (<1%)	0·99 (0·29–3·41)

Missing data are <1% unless otherwise stated.

* After the CORONIS birth and before any subsequent pregnancy, any new onset or worsening.

† Not related to pregnancy.

‡ Excluding diagnostic laparoscopy and diagnostic laparotomy.

§ After the CORONIS birth, any new onset.

**Table 5:** Outcomes associated with uterine repair sutures

		**Catgut**	**Polyglactin-910**	**Adjusted risk ratio (95% CI)**
Women's health				
	Denominator, women eligible to be assessed	4567	4564	
	Known deaths	22/4567 (<1%)	28/4564 (1%)	0·79 (0·45–1·37)
Subsequent pregnancies				
	Denominator, babies of women with a subsequent viable pregnancy*	1686	1670	
	Stillbirth†	43/1686 (3%)	33/1670 (2%)	1·29 (0·82–2·02)
	Neonatal death†‡	27/1643 (2%)	28/1634 (2%)	0·96 (0·57–1·61)
Mode of delivery†				
	Non-instrumental vaginal	190/1685 (11%)	199/1670 (12%)	..
	Instrumental vaginal	23/1685 (1%)	23/1670 (1%)	..
	Pre-labour caesarean section	1237/1685 (73%)	1226/1670 (73%)	..
	In-labour caesarean section	235/1685 (14%)	222/1670 (13%)	..
All caesarean sections		1472/1685 (87%)	1448/1670 (87%)	1·01 (0·98–1·03)
Denominator, women with a subsequent viable pregnancy*		1661	1648	
Other pregnancy complications, composite†§		42/1660 (2·5%)	35/1646 (2%)	1·19 (0·76–1·86)
	Uterine rupture†	3/1660 (<1%)	1/1647 (<1%)	3·05 (0·32–29·29)
	Uterine scar dehiscence†	2/1660 (<1%)	3/1646 (<1%)	0·67 (0·11–4·02)
	Placenta praevia†	8/1660 (<1%)	8/1646 (<1%)	1·00 (0·37–2·64)
	Morbidly adherent placenta†	2/1660 (<1%)	3/1646 (<1%)	0·68 (0·11–4·06)
	Abruption†	6/1660 (<1%)	2/1647 (<1%)	2·98 (0·60–14·74)
	Post-partum haemorrhage requiring transfusion of >1 unit of whole blood or packed cells†	8/1660 (<1%)	7/1647 (<1%)	1·14 (0·41–3·12)
	Severe infection within 6 weeks post partum†	16/1660 (1%)	9/1647 (<1%)	1·74 (0·77–3·94)
	Hysterectomy up to 6 weeks post partum†	1/1661 (<1%)	2/1647 (<1%)	0·50 (0·05–5·53)
	Manual removal of placenta†	5/1661 (<1%)	7/1647 (<1%)	0·71 (0·23–2·24)

Missing data are <1% unless otherwise stated.

* Viability could not be assessed if gestational age was not recorded.

† For the birth following the CORONIS birth.

‡ Excludes stillbirths.

§ Includes: uterine rupture, uterine scar dehiscence, placenta praevia, morbidly adherent placenta, abruption, post-partum haemorrhage requiring transfusion, severe infection within 6 weeks post-partum hysterectomy up to 6 weeks post partum, manual removal of placenta.
