# Supplementary material for: Caesarean section surgical techniques: 3 year follow-up of the CORONIS fractional, factorial, unmasked, randomised controlled trial
Source: Lancet. 2016 Jul 2;388(10039):62–72. doi: 10.1016/S0140-6736(16)00204-X (PMC4930950; doi:10.1016/S0140-6736(16)00204-X)
Supplement: Supplementary appendix [file mmc1.pdf]

THE LANCET

Supplementary appendix

This appendix formed part of the original submission and has been peer reviewed. We post it as supplied by the authors.

Supplement to: The CORONIS collaborative group. Caesarean section surgical techniques: 3 year follow-up of the CORONIS fractional, factorial, unmasked, randomised controlled trial. *Lancet* 2016; published online May 4. http://dx.doi.org/10.1016/S0140-6736(16)00204-X.

Supplementary material

This web appendix has been provided by the authors to give readers additional information about their work.

Supplement to Caesarean section surgical techniques and long term outcomes: follow-up of the CORONIS trial

The CORONIS Trial Collaborative Group

Contents

Defining the main comparisons of interest	3
Table S1 Comparisons of interest	4
Table S2 Settings	5
Table S3 Deaths by cause	6
Table S4 Characteristics of women assessed	7
Table S5 Comparability of women included with those not included	9
Table S6 Abdominal entry	10
Table S7 Closure of uterus	11
Table S8 Uterine repair sutures	12
Table S9 - Table S13 Secondary comparisons of interest	13
Table S14 All outcomes by sub-continent and overall	23
Table S15 Incidence of all outcomes by region and overall	25

Defining the main comparisons of interest

A 2x2x2x2x2 fractional factorial design is efficient because it can answer a range of questions with a smaller overall sample size then would be necessary if the questions were addressed individually. However, there are concerns about the number of comparisons undertaken when each intervention pair may have different outcomes of interest. For the main trial there was one overall primary outcome for all the intervention pairs. For the follow up study, there were likely to be differences in the impact of the different intervention pairs on a range of long term outcomes. For example, those intervention pairs which were concerned with the uterine scar incision, i.e. single versus double layer closure, or use of chromic catgut versus polyglactin-910, will most likely impact on outcomes associated with integrity of the uterine scar, such as uterine rupture or abnormal placentation. Whereas intervention pair such as closure versus non closure of the peritoneum may be more likely to impact on outcomes associated with intraperitoneal adhesions, such as pelvic pain, dyspareunia and infertility.

All potential outcomes associated with all the different techniques were initially enumerated. At a meeting of the CORONIS follow up study investigators in October 2010, an extensive exercise was undertaken to define which outcomes were most likely to be affected by the five intervention pairs based on the biological plausibility of seeing an impact. It was recognised that there were specific outcomes which were of major interest in evaluating the long term impact of specific intervention pairs, and these were labelled as main comparisons of interest, (M) in table S1. In addition there were outcomes which were less likely or which were less relevant but which were still of interest, and these were labelled as secondary comparisons of interest, (s) in table S1. And there were outcomes which were felt to be irrelevant to specific intervention pairs and these are the empty cells in table S1.

The process by which these comparisons were agreed was one of consensus development over a two day meeting between the clinicians amongst the co-investigator group. Alongside this exercise, a further exercise was undertaken once there was final agreement on the main comparisons of interest, to explore where there may be biologically plausible two-way interactions between intervention pairs focusing exclusively on the main comparisons of interest. It was initially agreed, as it was for the main trial, that no interactions would be explored unless there was evidence of a main effect. However, if there was evidence of a main effect, then biologically plausible interactions would be explored in the analysis.

Table S1 Comparisons of interest

Outcome reference number		Outcome	Blunt vs. Sharp	Exterior vs. Intra-abdominal	Single vs. Double	Closure vs. Non-Closure	Catgut vs. PG910
Women's health and mortality							
1	i)	Pelvic pain	s	s	s	M[a]	s[b]
	ii)	Dysmenorrhoea	s	s	s	s	s
	iii)	Deep dyspareunia	s	s	s	**M**	s
	iv)	Urinary symptoms	s	s	s	s	s
2		Diagnostic laparoscopy or diagnostic laparotomy - not related to pregnancy	s	s	s	**M**	s
3		Hysterectomy or tubal/ovarian surgery - not related to pregnancy	s	s	s	**M**	s
4		Bladder or bowel damage – not related to pregnancy	s	s	s	**M**	s
5	i)	Abdominal hernias	**M**			s	
	ii)	Bowel obstruction	s	s	s	**M**	s
6		Woman's death		s	**M**	s	**M**
Reproductive status							
7	i)	Women with no subsequent pregnancy – voluntary	s			s	
	ii)	Women with no subsequent pregnancy – involuntary	s	**M**	s	**M**	s
8		Fertility treatments	s	**M**	s	**M**	s
Subsequent pregnancies							
9		Any subsequent pregnancy	s	s	s	s	s
	i)	Interpregnancy interval	s	s	s	s	s
	ii)	Miscarriage		s	s		s
	iii)	Ectopic pregnancy		**M**	s	**M**	s
	iv)	Gestation		s	s		s
	v)	Stillbirth		s	**M**		**M**
	vi)	Neonatal death		s	**M**		**M**
	vii)	Mode of delivery		s	**M**		**M**
	viii)	Other pregnancy complications (composite of a-i)		s	**M**		**M**
	a	Uterine rupture		s	**M**		**M**
	b	Uterine scar dehiscence		s	**M**		**M**
	c	Placenta praevia		s	**M**		**M**
	d	Morbidly adherent placenta		s	**M**		**M**
	e	Abruption		s	**M**		**M**
	f	Postpartum haemorrhage requiring transfusion of >1 unit of whole blood or packed cells		s	**M**		**M**
	g	Severe infection within 6 weeks postpartum		s	**M**		**M**
	h	Hysterectomy up to 6 weeks postpartum		s	**M**		**M**
	i	Manual removal of placenta		s	**M**		**M**
	ix)	Bladder or bowel damage – at the time of subsequent Caesarean section	s	s	s	s	s
CORONIS children – morbidity and mortality							
10		Death or serious morbidity of child	**M**				

[a]M = Main comparison
[b]S = Secondary comparison

Settings

The CORONIS Trial and Follow-up Study was conducted in seven countries (8 regions) in 19 sites. The sites were a mixture of public and private hospitals, small and large and took both emergency and elective admissions. The numbers of women eligible to be assessed, and the number assessed are shown in Table S2.

Table S2 Settings

Regional Trial Offices	Eligible for follow-up[ab]	HAQs[c] received	Deaths[d]	Total	%
**Argentina**					
Hospital Dr Jose Maria Cullen	200	166		166	83
Hospital Interzonal General de Agudos Dr Jose Penna	505	390	1	391	77
Hospital J.B. Iturraspe	237	179	1	180	76
Hospital Nacional Profesor Alejandro Posadas	431	323	2	325	75
Hospital Regional Dr Ramon Carillo	243	213	1	214	88
**Total for Argentina**	**1616**	**1271**	**5**	**1276**	**79**
**Chile**					
Pontificia Universidad Católica de Chile Hospital	505	486	0	486	96
Sotero Del Rio Hospital	700	648	3	651	93
**Total for Chile**	**1205**	**1134**	**3**	**1137**	**94**
**Ghana**					
Komfo Anokye Teaching Hospital	**1260**	**1041**	**3**	**1044**	**83**
**India -Delhi**					
All India Institute of Medical Sciences (AIIMS)	565	478	6	484	86
Lady Hardinge Medical College & Sucheta Kriplani Hospital	1062	819	5	824	78
Maulana Azad Medical College & Lok Nayak Hospital	1027	729	6	735	72
University College of Medical Sciences & GTB Hospital	1100	836	8	844	77
**Total for Delhi**	**3754**	**2862**	**25**	**2887**	**77**
**Kenya**					
Kenyatta National Hospital	**1653**	**1446**	**14**	**1461**	**88**
**Pakistan**					
Countess of Dufferin Hospital	1493	1191	4	1195	80
Fatima Bai Hospital	468	371	3	374	80
Liaquat National Hospital	563	476	1	477	85
**Total for Pakistan**	**2524**	**2038**	**8**	**2046**	**81**
**Sudan**					
Omdurman Maternity Hospital	1056	780	4	784	74
Soba University Hospital	537	518	2	520	97
**Total for Sudan**	**1593**	**1298**	**6**	**1304**	**82**
**India - Vellore**					
Christian Medical College Hospital	**2101**	**2063**	**9**	**2072**	**99**
**Overall**	**15706**	**13153**	**73**	**13227**	**84**

[a] Excludes women known to be dead at six weeks, women randomised in error, women who withdrew consent from main trial, baseline data not received, vaginal deliveries at the time of recruitment to CORONIS.
[b] Includes women eligible to be contacted for follow-up and assessment
[c] HAQ = Health Assessment Questionnaire
[d] Deaths reported during the follow-up study

Table S3 Deaths by cause

This table includes all known deaths of women participating in CORONIS from the time of recruitment into the trial until the time that follow-up was achieved.

Cause	Number of deaths						
	First year	Second year	Third year	Fourth year	Fifth year	Sixth year	Total
Pregnancy related[a]	8	1	1	0	0	1	11
Infection[b]	8	2	2	2	2	2	18
Cancer	4	1	0	1	1	0	7
Other medical[c]	6	7	7	6	4	0	30
Suicide/Accident[d]	2	3	2	2	1	0	10
Unknown	5	2	2	3	0	1	13
	**33**	**16**	**14**	**14**	**8**	**4**	**89**

[a] Includes hypertensive disease, Mendelson's syndrome, haemorrhage, PIH, DIC
[b] Includes HIV/AIDS, Hepatitis C, tuberculosis, H1N1 influenza
[c] Includes epilepsy, stroke, liver failure, diabetes, heart disease, renal failure, Crohns disease, heart disease
[d] Suicide has been included with accidental death, as this is how these deaths are reported in some of these settings

Table S4 Characteristics of women assessed

	Abdominal entry		Repair of uterus		Closure of uterus		Closure of peritoneum		Uterine repair sutures		Total
	Blunt N = 3878	Sharp N = 3873	Exterior N = 4092	Intra-ab N = 4136	Single N = 3709	Double N = 3702	Closure N = 4036	Non-closure N = 4087	Catgut N = 3974	PG-910 N = 3972	N = 13153
**Region**											
Argentina	635 (16)	636 (16)	629 (15)	642 (16)	N/A[a]	N/A[a]	642 (16)	629 (15)	N/A[a]	N/A[a]	1271 (10)
Chile	N/A[a]	N/A[a]	567 (14)	567 (14)	325 (9)	323 (9)	561 (14)	573 (14)	246 (6)	240 (6)	1134 (9)
Ghana	516 (13)	525 (14)	N/A[a]	N/A[a]	510 (14)	531 (14)	N/A[a]	N/A[a]	531 (13)	510 (13)	1041 (8)
India – Delhi	1016 (26)	1010 (26)	825 (20)	830 (20)	1436 (39)	1426 (39)	1010 (25)	1033 (25)	N/A[a]	N/A[a]	2862 (22)
India – Vellore[b]	772 (20)	769 (20)	261 (6)	261 (6)	N/A[a]	N/A[a]	1028 (25)	1035 (25)	1033 (26)	1030 (26)	2063 (16)
Kenya	728 (19)	718 (19)	726 (18)	720 (17)	N/A[a]	N/A[a]	N/A[a]	N/A[a]	724 (18)	722 (18)	1446 (11)
Pakistan[b]	211 (5)	215 (6)	439 (11)	463 (11)	777 (21)	785 (21)	795 (20)	817 (20)	807 (20)	805 (20)	2038 (15)
Sudan	N/A[a]	N/A[a]	645 (16)	653 (16)	661 (18)	637 (17)	N/A[a]	N/A[a]	633 (16)	665 (17)	1298 (10)
**Maternal characteristics**											
Mother's age - mean [SD]	26·7 [5·2]	26·9 [5·3]	27·3 [5·7]	27·4 [5·7]	27·1 [5·4]	27·0 [5·4]	26·6 [5·2]	26·7 [5·3]	28·1 [5·3]	28·1 [5·4]	27·2 [5·4]
Nulliparous[c]	1991 (51)	1947 (50)	1832 (45)	1875 (45)	1733 (47)	1780 (48)	1911(47)	1955 (48)	1735 (44)	1733 (44)	6164 (47)
Previous CS											
None	2776 (72)	2781 (72)	2689 (66)	2708 (65)	2524 (68)	2496 (67)	2567 (64)	2589 (63)	2517 (63)	2489 (63)	8712 (66)
One	1102 (28)	1092 (28)	1403 (34)	1428 (35)	1185 (32)	1206 (33)	1469 (36)	1498 (37)	1457 (37)	1483 (37)	4441 (34)
Previous CS incision											
Abdominal transverse	1051 (95)	1041 (95)	1364 (97)	1403 (98)	1155 (97)	1176 (98)	1413 (96)	1440 (96)	1401 (96)	1426 (96)	4290 (97)
Uterine transverse	1035 (94)	1029 (94)	1246 (89)	1261 (88)	1030 (87)	1052 (87)	1340 (91)	1365 (91)	1384 (95)	1417 (96)	4053 (91)
Uterine unknown	62 (6)	60 (5)	151 (11)	160 (11)	150 (13)	149 (12)	125 (9)	126 (8)	69 (5)	61 (4)	371 (8)
HIV status											
Known positive	124 (3)	124 (3)	104 (3)	110 (3)	31 (1)	22 (1)	14 (<1)	20 (<1)	115 (3)	107 (3)	257 (2)
Known negative	2825 (73)	2847 (74)	2495 (61)	2503 (61)	1463 (39)	1451 (39)	2867 (71)	2871 (70)	2046 (51)	2026 (51)	7798 (59)
Status unknown	929 (24)	902 (23)	1493 (36)	1523 (37)	2215 (60)	2229 (60)	1155 (29)	1196 (29)	1813 (46)	1839 (46)	5098 (39)
Fetal heart beat heard	3850 (99)	3848 (99)	4075 (99)	4114 (99)	3694 (99)	3688 (99)	4027 (99)	4084 (99)	3943 (99)	3947 (99)	13090 (99)
Planned VBAC[d]	142/110 (13)	179/1092 (16)	147/1403 (11)	149/1428 (10)	109/1185 (9)	131/1206 (11)	175/1469 (12)	184/1498 (12)	149/1457 (10)	141/1483 (10)	502/4441 (11)
Ruptured membranes before delivery	1444 (37)	1422 (37)	1146 (28)	1138 (28)	1036 (28)	1027 (28)	1239 (31)	1297 (32)	1193 (30)	1178 (30)	4040 (31)
Labour prior to CS	1966 (51)	1957 (51)	1659 (41)	1665 (40)	1599 (43)	1572 (42)	1769 (44)	1795 (44)	1547 (39)	1556 (39)	5695 (43)
Duration of labour											
< 6 hours	651 (33)	663 (34)	556 (34)	548 (33)	574 (36)	555 (35)	705 (40)	695 (39)	434 (28)	412 (26)	1931 (34)
6-12 hours	792 (40)	743 (38)	724 (44)	741 (45)	617 (39)	602 (38)	727 (41)	769 (43)	608 (39)	637 (41)	2320 (41)
> 12 hours	298 (15)	325 (17)	288 (17)	256 (15)	206 (13)	216 (14)	305 (17)	302 (17)	296 (19)	289 (19)	927 (16)
Duration unknown	225 (11)	226 (12)	91 (5)	120 (7)	202 (13)	199 (13)	32 (2)	29 (2)	209 (14)	218 (14)	517 (9)

Intrapartum fever ≥ 38°C any											
time during labour[e]											
Unknown	39 (2)	41 (2)	30 (2)	23 (1)	28 (2)	35 (2)	40 (2)	36 (2)	42 (3)	37 (2)	117 (2)
	40 (2)	39 (2)	10 (1)	10 (1)	34 (2)	43 (3)	3 (<1)	4 (<1)	45 (3)	36 (2)	88 (2)
CS undertaken											
before start of labour	1912 (49)	1916 (49)	2433 (59)	2471 (60)	2110 (57)	2130 (58)	2267 (56)	2292 (56)	2427 (61)	2416 (61)	7458 (57)
after start of labour	1966 (51)	1957 (51)	1659 (41)	1665 (40)	1599 (43)	1572 (42)	1769 (44)	1795 (44)	1547 (39)	1556 (39)	5695 (43)
Main indication for CS											
Fetal compromise	973 (25)	954 (25)	799 (20)	817 (20)	796 (21)	799 (22)	905 (22)	917 (22)	778 (20)	725 (18)	2821 (21)
Failure to progress	842 (22)	828 (21)	841 (21)	802 (19)	896 (24)	855 (23)	911 (23)	898 (22)	877 (22)	884 (22)	2878 (22)
Obst. complications	1287 (33)	1301 (34)	1317 (32)	1384 (33)	1321 (36)	1322 (36)	1215 (30)	1246 (30)	1217 (31)	1245 (31)	4285 (33)
Maternal request	188 (5)	166 (4)	220 (5)	171 (4)	84 (2)	89 (2)	247 (6)	260 (6)	278 (7)	298 (8)	667 (5)
Previous C-section	256 (7)	278 (7)	405 (10)	419 (10)	194 (5)	225 (6)	459 (11)	445 (11)	258 (6)	256 (6)	1065 (8)
Other	332 (9)	346 (9)	510 (12)	543 (13)	418 (11)	412 (11)	299 (7)	321 (8)	566 (14)	564 (14)	1437 (11)
Anaesthetic used for CS											
Regional											
General	3364 (87)	3371 (87)	3695 (90)	3745 (91)	3490 (94)	3466 (94)	3881 (96)	3930 (96)	3417 (86)	3434 (86)	11931 (91)
	514 (13)	502 (13)	397 (10)	391 (9)	219 (6)	236 (6)	155 (4)	157 (4)	557 (14)	538 (14)	1222 (9)
Gestational age at birth[f]											
<32											
32-36	57 (1)	53 (1)	42 (1)	46 (1)	29 (1)	37 (1)	49 (1)	47 (1)	36 (1)	51 (1)	149 (1)
37-41	514 (13)	548 (14)	452 (11)	443 (11)	571 (15)	540 (15)	608 (15)	628 (15)	442 (11)	471 (12)	1739 (13)
> 41	3198 (82)	3172 (82)	3544 (87)	3568 (86)	3014	3020 (82)	3357 (83)	3378 (83)	3395 (85)	3342 (84)	10996 (84)
	89 (2)	90 (2)	49 (1)	67 (2)	81 (2)	92 (2)	21 (1)	32 (1)	87 (2)	94 (2)	234 (2)
**Associated information**											
Time from trial entry to surgery											
(mins)-Med {IQR}	29 {17-54}	29 {17-51}	33 {18-74}	32 {17-74}	28 {14-59}	28 {14-58}	29 {16-59}	29 {16-61}	28 {15-55}	28 {16-56}	29 {16-60}
> 4 hours[g]	152 (4)	112 (3)	235 (6)	243 (6)	185 (5)	195 (5)	208 (5)	207 (5)	106 (3)	133 (3)	592 (5)
Surgeon's experience (yrs) -											
Median {IQR)	5 {4-8}	5 {4-8}	7 {4-13}	7 {4-13}	8 {5-14}	8 {5-14}	6 {4-12}	6 {4-12}	7 {4-13}	7 {4-13}	6 {4-12}
<5	1493 (39)	1491 (39)	1255 (31)	1273 (31)	814 (22)	819 (22)	1331 (33)	1362 (33)	1072 (27)	1072 (27)	3994 (30)
5 to 10	1676 (43)	1652 (43)	1349 (33)	1391 (34)	1290 (35)	1304 (35)	1430 (35)	1426 (35)	1384 (35)	1363 (34)	4755 (36)
>10	691 (18)	703 (18)	1468 (36)	1454 (35)	1580 (43)	1559 (42)	1273 (32)	1294 (32)	1518 (38)	1537 (39)	4359 (33)

[a] Intervention pairs not assigned in these regions.
[b] Some sites within these regions switched intervention pairs.
[c] Nulliparous women are women with no previous births. A previous birth is a live or stillbirth of estimated gestational age > 24 weeks (or > 28 weeks depending on country-specific definitions) regardless of previous mode of delivery or multiple pregnancy (multiple pregnancy counts as one birth).
[d]VBAC - Vaginal Birth After Caesarean section, described in the main trial as 'trial of scar'.
[e] Denominator is women with labour diagnosed before CS.
[f] Completed weeks.
[g] In some sites planned elective CS were randomised the evening before surgery.

Table S5 Comparability of women included in the analysis with those not included

	Included	Not included	p-value
**Population – women assessed**	**13153**	**2570**	
Mother's age – mean [sd]	27·2 [5·4]	26·0 [5·4]	<0·001
Nulliparous	6164 / 13153 (46·9%)	1220 / 2570 (47·5%)	0·57
1 previous caesarean section	4441 / 13153 (33·8%)	722 / 2570 (28·1)	<0·001
Caesarean section before start of labour	7458 / 13153 (56·7%)	1281 / 2570 (49·8)	<0·001
Short-term primary outcome[a]	1227 / 13153 (9·3%)	240 / 2568 (9·3%)	0·98
Still birth or death of CORONIS baby by 6 weeks of age	412 / 13053 (3·2%)	115 / 2389 (4·8%)	<0·001

[a] A woman may have more than one component but is included only once in the primary outcome. Primary outcome defined as death or maternal infectious morbidity or further operative procedures or blood transfusion (>1 unit of whole blood or packed cells).

Table S6 **Abdominal entry**

Secondary analysis, including stillbirths with death or serious morbidity (see Table 2 in main paper)

Outcome	Blunt	Sharp	Adjusted RR (95% CI)
CORONIS child(ren) **Denominator –children of women eligible to be assessed**	**4779**	**4817**	
Known stillbirths, deaths or serious morbidity of CORONIS child(ren)[a]	298 / 4779 (6·2%)	294 / 4817 (6·1%)	02 (0·87 to 1·19)

[a] Includes stillbirths

Table S7 Closure of uterus

Secondary analysis where the denominator is all women assessed, not only those with a subsequent viable pregnancy (see Table 4 in main paper).

	Single	Double	Adjusted risk ratio (95% CI)
Subsequent pregnancies			
**Denominator – babies of women assessed**	**3728**	**3724**	
Stillbirth[a]	34 / 3712 (0·9%)	28 / 3709 (0·8%)	1·21 (0·74 to 2·00)
Neonatal death[ab]	32 / 3677 (0·9%)	34 / 3679 (0·9%)	0·94 (0·58 to 1·53)
Mode of delivery[a]			
All caesarean sections	1312 / 3712 (35·3%)	1353 / 3709 (36·5%)	0·97 (0·91 to 1·03)
**Denominator – women assessed**	**3709**	**3702**	
Other pregnancy complications – composite[ac]	38 / 3691(1·0%)	32 / 3686 (0·9%)	1·19 (0·74 to 1·89)
Uterine rupture[a]	1 / 3692 (0·03%)	2 / 3687 (0·05%)	0·50 (0·04 to 5·46)
Uterine scar dehiscence[a]	4 / 3691 (0·11%)	2 / 3687 (0·05%)	1·99 (0·37 to 10·88)
Placenta praevia[a]	5 / 3691 (0·1%)	4 / 3687 (0·1%)	1·25 (0·34 to 4·65)
Morbidly adherent placenta[a]	0 / 3691 (0·00%)	2 / 3687 (0·05%)	-
Abruption[a]	6 / 3692 (0·2%)	4 / 3687 (0·1%)	1·49 (0·42 to 5·27)
Postpartum haemorrhage requiring transfusion of >1 unit of whole blood or packed cells[a]	8 / 3692 (0·2%)	7 / 3687 (0·2%)	1·14 (0·41 to 3·15)
Severe infection within 6 weeks postpartum[a]	14 / 3692 (0·4%)	15 / 3686 (0·4%)	0·93 (0·45 to 1·93)
Hysterectomy up to 6 weeks postpartum[a]	1 / 3692 (0·03%)	1 / 3687 (0·03%)	1·00 (0·06 to 16·04)
Manual removal of placenta[a]	4 / 3692 (0·1%)	0 / 3687 (0·0%)	-

[a] For the birth following the CORONIS birth.
[b] Excludes stillbirths.
[c] Includes: uterine rupture, uterine scar dehiscence, placenta praevia, morbidly adherent placenta, abruption postpartum haemorrhage requiring transfusion, severe infection within 6 weeks, postpartum hysterectomy up to 6 weeks postpartum, manual removal of placenta.

Table S8 Uterine repair sutures

Secondary analysis where the denominator is all women assessed, not only those with a subsequent viable pregnancy (see Table 6 in main paper)

Outcome	Catgut	PG-910	Adjusted risk ratio (95% CI)
Subsequent pregnancies			
**Denominator – babies of women assessed**	**3999**	**3994**	
Stillbirth[a]	43 / 3982 (1·1%)	33 / 3985 (0·8%)	1·30 (0·83 to 2·04)
Neonatal death[ab]	27 / 3939 (0·7%)	28 / 3949 (0·7%)	0·97 (0·57 to 1·64)
Mode of delivery[a]			
All caesarean sections	1472 / 3981 (37·0%)	1448 / 3985 (36·3%)	1·01 (0·96 to 1·08)
**Denominator – women assessed**	**3974**	**3972**	
Other pregnancy complications – composite[ac]	42 / 3956 (1·1%)	35 / 3961 (0·9%)	1·20 (0·77 to 1·88)
Uterine rupture[a]	3 / 3956 (0·08%)	1 / 3962 (0·03%)	3·01 (0·31 to 28·96)
Uterine scar dehiscence[a]	2 / 3956 (0·05%)	3 / 3961 (0·08%)	0·67 (0·11 to 4·01)
Placenta praevia[a]	8 / 3956 (0·2%)	8 / 3961 (0·2%)	1·01 (0·38 to 2·68)
Morbidly adherent placenta[a]	2 / 3956 (0·05%)	3 / 3961 (0·08%)	0·67 (0·11 to 4·01)
Abruption[a]	6 / 3956 (0·2%)	2 / 3962 (0·1%)	2·99 (0·60 to 14·80)
Postpartum haemorrhage requiring transfusion of >1 unit of whole blood or packed cells[a]	8 / 3956 (0·2%)	7 / 3962 (0·2%)	1·14 (0·42 to 3·16)
Severe infection within 6 weeks postpartum[a]	16 / 3956 (0·4%)	9 / 3962 (0·2%)	1·77 (0·78 to 4·00)
Hysterectomy up to 6 weeks postpartum[a]	1 / 3957 (0·03%)	2 / 3962 (0·05%)	0·50 (0·05 to 5·56)
Manual removal of placenta[a]	5 / 3957 (0·1%)	7 / 3962 (0·2%)	0·71 (0·23 to 2·24)

[a] For the birth following the CORONIS birth.
[b] Excludes stillbirths.
[c] Includes: uterine rupture, uterine scar dehiscence, placenta praevia, morbidly adherent placenta, abruption, postpartum haemorrhage requiring transfusion, severe infection within 6 weeks, postpartum hysterectomy up to 6 weeks postpartum, manual removal of placenta.

Tables S9 to S12 Secondary comparisons of interest by allocation

Table S9 Abdominal entry

Outcome	Blunt	Sharp	Adjusted risk ratio (99% CI)
Women's health			
**Denominator – women assessed**	**3878**	**3873**	
Pelvic pain[a]	156 / 3878 (4·0%)	136 / 3873 (3·5%)	1·14 (0·85 to 1·54)
Dysmenorrhoea[a]	179 / 3878 (4·6%)	176 / 3873 (4·5%)	1·02 (0·78 to 1·33)
Deep dyspareunia[a]	118 / 3878 (3·0%)	112 / 3873 (2·9%)	1·05 (0·75 to 1·47)
Urinary symptoms[a b]	39 / 3878 (1·0%)	35 / 3873 (1·0%)	1·11 (0·61 to 2·02)
Diagnostic laparoscopy/laparotomy[c]	0 / 3878 (0·0%)	3 / 3871 (0·08%)	-
Hysterectomy or tubal/ovarian surgery[c]	9 / 3878 (0·2%)	10 / 3873 (0·3%)	0·91 (0·28 to 2·97)
Bladder or bowel damage following surgery[cd]	0 / 75 (0·0%)	1 / 64 (1·6%)	-
Bowel obstruction[e]	0 / 3878 (0·0%)	3 / 3873 (0·08%)	-
Reproductive status			
**Denominator - Women with no subsequent pregnancy**	**2266**	**2236**	
Voluntary infertility	2144 / 2266 (94·6%)	2099 / 2236 (94·0%)	1·00 (0·99 to 1·03)
**Denominator – women assessed**	**3878**	**3873**	
Voluntary infertility	2144 / 3878 (55·3%)	2099 / 3873 (54·2%)	1·02 (0·97 to 1·07)
Involuntary infertility	98 / 3878 (2·5%)	116 / 3873 (3·0%)	0·86 (0·10 to 1·21)
Use of fertility treatments	12 / 3877 (0·3%)	14 / 3873 (0·4%)	0·86 (0·31 to 2·35)

Table S9 continued

Outcome	Blunt	Sharp	Adjusted risk ratio or hazards ratio (HR) (99% CI)
Subsequent pregnancies			
**Denominator – women assessed**	**3878**	**3873**	
Any subsequent pregnancy	1612 / 3878 (41·6%)	1637 / 3873 (42·3%)	0·99 (0·92 to 1·05)
**Denominator – women with a subsequent pregnancy**	**1612**	**1637**	
Interpregnancy interval (months) [f]			
Mean [sd]	30·2 [12·88]	30·5 [12·46]	HR
Median {IQR}	28·7 {20·5 to 38·0}	29·5 {21·3 to 38·3}	1·00 (0·92 to 1·10)
**Denominator - women with a subsequent viable pregnancy**	**1367**	**1396**	
Bladder or bowel damage[j]	1 / 1105 (0·1%)	1 / 1132 (0·1%)	1·02 (0·03 to 39·07)
**Denominator - women assessed**	**3878**	**3873**	
Bladder or bowel damage[j]	1 / 3595 (0·03%)	1 / 3592 (0·03%)	1·00 (0·03 to 38·15)

[a] Following the CORONIS birth and before any subsequent pregnancy, any new onset or worsening.
[b] Urinary symptoms of poor stream and/or frequency which did not respond to antibiotics.
[c] Not related to pregnancy.
[d] In those women who have had surgery excluding diagnostic laparoscopy and diagnostic laparotomy.
[e] Following the CORONIS birth any new onset.
[f] From the CORONIS birth to the end of the subsequent pregnancy (regardless of loss or birth·)
[g] Of the first viable pregnancy (gestational age > 24 or >28 weeks depending on country specific definition.
[h] For the birth following the CORONIS birth.
[i] Includes: uterine rupture, uterine scar dehiscence, placenta praevia, morbidly adherent placenta, abruption, postpartum haemorrhage requiring transfusion, severe infection within 6 weeks, postpartum hysterectomy up to 6 weeks postpartum, manual removal of placenta.
[j] At the time of subsequent Caesarean section.

Table S10 Repair of uterus

Outcome	Exterior	Intra-ab	Adjusted risk ratio or hazards ratio (HR) (99% CI)
Women's health			
**Denominator – women assessed**	**4092**	**4136**	
Pelvic pain[a]	223 / 4092 (5·5%)	234 / 4136 (5·7%)	0·97 (0·76 to 1·22)
Dysmenorrhoea[a]	246 / 4092 (6·0%)	274 / 4136 (6·6%)	0·91 (0·73 to 1·13)
Deep dyspareunia[a]	156 / 4092 (3·8%)	173 / 4136 (4·2%)	0·91 (0·69 to 1·20)
Urinary symptoms[a·b]	42 / 4092 (1·0%)	55 / 4136 (1·3%)	0·77 (0·46 to 1·30)
Diagnostic laparoscopy/laparotomy[c]	1 / 4092 (0·02%)	6 / 4136 (0·15%)	0·17 (0·01 to 2·73)
Hysterectomy or tubal/ovarian surgery[c]	7 / 4092 (0·2%)	13/ 4136 (0·3%)	0·54 (0·16 to 1·82)
Bladder or bowel damage[cd]	0 / 89 (0·0%)	1 / 98 (1·02%)	-
Bowel obstruction[e]	3 / 4092 (0·07%)	2 / 4136 (0·05%)	1·51 (0·14 to 15·88)
**Denominator – women eligible to be assessed**	**4867**	**4862**	
Woman's death	26 / 4867 (0·5%)	23 / 4862 (0·5%)	1·13 (0·54 to 2·36)
Subsequent pregnancies			
**Denominator – women assessed**	**4092**	**4136**	
Any subsequent pregnancy	1653 / 4092 (40·4%)	1656 / 4136 (40·0%)	1·00 (0·94 to 1·08)
**Denominator – women with a subsequent pregnancy**	**1653**	**1656**	
Interpregnancy interval (months)[f]			
Mean [sd]	29·3 [11·8]	29.6 [12.0]	HR
Median {IQR}	28·2 {20·5 to 36·4}	28·8 {20·6 to 37·1}	1·04 (0·95 to 1·13)
Miscarriage	205 / 1627 (12·6%)	204 / 1632 (12·5%)	1·00 (0·80 to 1·28)

Table S10 continued

Outcome	Exterior	Intra-ab	Adjusted risk ratio or mean difference (MD) (99% CI)
**Denominator - women with a subsequent viable pregnancy**	**1407**	**1413**	
Gestation at delivery (completed wks)[g]			MD
Mean [sd]	38·3 [1·5]	38·3 [1·6]	-0·06 (-0·21 to 0·09)
Median {IQR}	38·6 {38·0 to 38·6}	38·6 {38·0 to 39·0}	
**Denominator – babies of women with a subsequent viable pregnancy**	**1423**	**1425**	
Stillbirth[h]	23 / 1423 (1·6%)	20 / 1425 (1·4%)	1·15 (0·52 to 2·50)
Neonatal death[i]	25 / 1400 (1·8%)	23 / 1405 (1·6%)	1·10 (0·52 to 2·30)
Mode of delivery[h]			
Non-instrumental vaginal	212/1423 (14·9%)	206/1425 (14·5%)	
Instrumental vaginal	6/1423 (0·4%)	6/1425 (0·4%)	
Pre-labour caesarean section	941/1423 (66·2%)	957/1425 (67·2%)	
In labour caesarean section	263/1423 (18·5%)	256/1425 (17·9%)	
All caesarean sections	1204 / 1422 (84·7%)	1213 / 1425 (85·1%)	0.99 (0·95 to 1·03)
**Denominator - women with a subsequent viable pregnancy**	**1407**	**1413**	
Other pregnancy complications – composite[hi]	38 / 1407 (2·7%)	50 / 1410 (3·5%)	0·80 (0·44 to 1·32)
Uterine rupture[h]	1 / 1407 (0·07%)	1 / 1412 (0·07%)	0·97 (0·03 to 36·8)
Uterine scar dehiscence[h]	4 / 1407 (0·3%)	10 / 1412 (0·7%)	1·01 (0·20 to 6·22)
Placenta praevia[h]	4/ 1407 (0·3%)	10 / 1412 (0·7%)	0·40 (0·09 to 1·84)
Morbidly adherent placenta[h]	0 / 1407(0·0%)	4 / 1412 (0·3%)	-
Abruption[h]	5 / 1407 (0·4%)	4 / 1412 (0·3%)	1·25 (0·22 to 7·00)
Postpartum haemorrhage requiring transfusion of >1 unit of whole blood or packed cells[h]	7 / 1407 (0·5%)	9 / 1412 (0·6%)	0·75 (0·20 to 2·73)
Severe infection within 6 weeks postpartum[h]	17 / 1407 (1·2%)	14 / 1410 (1·0%)	1·21 (0·50 to 3·06)

Table S10 continued

Outcome	Exterior	Intra-ab	Adjusted risk ratio or mean difference (MD) (99% CI)
Hysterectomy up to 6 weeks postpartum[h]	1 / 1407 (0·1%)	3 / 1413 (0·2%)	0·34 (0·02 to 6·60)
Manual removal of placenta[h]	3 / 1407 (0·2%)	11 / 1413 (0·8%)	0·28 (0·05 to 1·5)
Bladder or bowel damage[j]	0 / 1190 (0·0%)	1 / 1203 (0·1%)	-
**Denominator – babies of women assessed**	**4082**	**4124**	
Stillbirth[h]	23 / 4082 (0·6%)	20 / 4124 (0·5%)	1·16 (0·53 to 2·55)
Neonatal death[i]	25 / 4059 (0·6%)	23 / 4104 (0·6%)	1·10 (0·52 to 2·31)
Mode of delivery[h] All caesarean sections	1204 /4081 (29·5%)	1213 / 4124 (29·4%)	1·00 (0·92 to 1·10)
**Denominator - women assessed**	**4092**	**4136**	
Other pregnancy complications - composite[hi]	38 / 4066 (0·9%)	50 / 4109 (1·2%)	0·77 (0·44 to 1·33)
Uterine rupture[h]	1 / 4066 (0·02%)	1 / 4111(0·02%)	1·01 (0·03 to 38·73)
Uterine scar dehiscence[h]	4 / 4066 (0·1%)	4 / 4111 (0·1%)	1·01 (0·16 to 6·24)
Placenta praevia[h]	4 / 4066 (0·1%)	10/ 4111 (0·2%)	0·40 (0·10 to 1·86)
Morbidly adherent placenta[h]	0 / 4066 (0·0%)	4 / 4111(0·1%)	-
Abruption[h]	5 / 4066 (0·1%)	4 / 4111 (0·1%)	1·3 (0·22 to 7·10)
Postpartum haemorrhage requiring transfusion of >1 unit of whole blood or packed cells[h]	7 / 4066 (0·02%)	9 / 4111 (0·02%)	0·80 (0·22 to 2·90)
Severe infection within 6 weeks postpartum[h]	17 / 4066 (0·4%)	14 / 4109 (0·3%)	1·22 (0·50 to 3·10)
Hysterectomy up to 6 weeks postpartum[h]	1 / 4066 (0·02%)	3 /4112 (0·07%)	0·34 (0·02 to 6·60)
Manual removal of placenta[h]	3 / 4066 (0·1%)	11 / 4111 (0·3%)	0·30 (0·51 to 1·50)
Bladder or bowel damage[j]	0 / 3849 (0·00%)	1 / 3902 (0·03%)	-

[a] Following the CORONIS birth and before any subsequent pregnancy, any new onset or worsening.
[b] Urinary symptoms of poor stream and/or frequency which did not respond to antibiotics.
[c] Not related to pregnancy.
[d] In those women who have had surgery excluding diagnostic laparoscopy and diagnostic laparotomy.
[e] Following the CORONIS birth any new onset.
[f] From the CORONIS birth to the end of the subsequent pregnancy (regardless of loss or birth·)
[g] Of the first viable pregnancy (gestational age > 24 or >28 weeks depending on country specific definition.
[h] For the birth following the CORONIS birth.
[i] Includes: uterine rupture, uterine scar dehiscence, placenta praevia, morbidly adherent placenta, abruption, postpartum haemorrhage requiring transfusion, severe infection within 6 weeks, postpartum hysterectomy up to 6 weeks postpartum, manual removal of placenta.
[j] At the time of subsequent Caesarean section.

Table S11 Closure of uterus

Outcome	Single	Double	Adjusted risk ratio, mean difference (MD) or hazards ratio (HR) (99% CI)
Women's health			
**Denominator – women assessed**	**3709**	**3702**	
Pelvic pain[a]	211 / 3709 (5·7%)	176 / 3702 (4·8%)	1·20 (0·93 to 1·55)
Dysmenorrhoea[a]	208 / 3709 (5·6%)	171 / 3702 (4·6%)	1·22 (0·94 to 1·57)
Deep dyspareunia[a]	110 / 3709 (3·0%)	93 / 3702 (2·5%)	1·19 (0·83 to 1·69)
Urinary symptoms[a b]	35 / 3709 (1·0%)	27 / 3702 (1·0%)	1·30 (0·67 to 2·51)
Diagnostic laparoscopy/laparotomy[c]	1 / 3707 (0·03%)	1 / 3702 (0·03%)	1·01 (0·03 to 38·31)
Hysterectomy or tubal/ovarian surgery[c]	4 / 3709 (0·1%)	4 / 3702 (0·1%)	1·00 (0·16 to 6·15)
Bladder or bowel damage[cd]	0 / 3709 (0·0%)	0 /3702 (0·0%)	-
Bowel obstruction[e]	1 / 3709 (0·03%)	1 / 3702 (0·03%)	1·00 (0·26 to 38·05)
Reproductive status			
**Denominator – women assessed**	**3709**	**3702**	
Involuntary infertility	133 / 3709 (3·6%)	132 / 3702 (3·6%)	1·01 (0·74 to 1·37)
Use of fertility treatments	20 / 3709 (0·5%)	10 / 3701 (0·3%)	1·98 (0·73 to 5·38)
Subsequent pregnancies			
**Denominator – women assessed**	**3709**	**3702**	
Any subsequent pregnancy	1889 / 3709 (51·0%)	1904 / 3702 (51·4%)	0·9 (0·93 to 1·05)
**Denominator – women with a subsequent pregnancy**	**1889**	**1904**	
Interpregnancy interval (months) [f]			
Mean [sd]	29·0 [11·6]	29·1 [11·7]	HR
Median {IQR}	27·9 {20·6 to 36·1}	28·2 {20·5 to 36·5}	1·01 (0·93 to 1·10)
Miscarriage	234 / 1873 (12·5%)	251 /1889 (13·3%)	0·94 (0·76 to 1·17)
Ectopic pregnancy	6 / 1873 (0·3%)	4 /1889 (0·2%)	1·51 (0·29 to 7·94)
**Denominator - women with a subsequent viable pregnancy**	**1889**	**1904**	
Gestation at delivery (completed wks)[g]			MD
Mean [sd]	38·2 [1·3]	38·1 [1·3]	0·04 (-0·07 to 0·16)
Median {IQR}	38·6 {38·0 to 38·6}	38·6 {38·0 to 38·6}	
Bladder or bowel damage[j]	1 / 1298 (0·08%)	0 / 1332 (0·00%)	-
**Denominator - women assessed**	**3709**	**3702**	
Bladder or bowel damage[j]	1 / 3709 (0·03%)	0 /3702 (0·00%)	-

[a] Following the CORONIS birth and before any subsequent pregnancy, any new onset or worsening.
[b] Urinary symptoms of poor stream and/or frequency which did not respond to antibiotics.
[c] Not related to pregnancy.

[d] In those women who have had surgery excluding diagnostic laparoscopy and diagnostic laparotomy.
[e] Following the CORONIS birth any new onset.
[f] From the CORONIS birth to the end of the subsequent pregnancy (regardless of loss or birth·)
[g] Of the first viable pregnancy (gestational age > 24 or >28 weeks depending on country specific definition.
[h] For the birth following the CORONIS birth.
[i] Includes: uterine rupture, uterine scar dehiscence, placenta praevia, morbidly adherent placenta, abruption, postpartum haemorrhage requiring transfusion, severe infection within 6 weeks, postpartum hysterectomy up to 6 weeks postpartum, manual removal of placenta.
[j] At the time of subsequent Caesarean section.

Table S12 Closure of peritoneum

Outcome	Closure	Non-Closure	Adjusted risk ratio or hazards ratio (HR) (99% CI)
Women's health and mortality			
**Denominator – women assessed**	**4036**	**4087**	
Dysmenorrhoea[a]	255 / 4306 (6·3%)	235 / 4087 (5·7%)	1·10 (0·88 to 1·38)
Urinary symptoms[a,b]	39 / 4036 (1·0%)	51 / 4087 (1·2%)	0·77 (0·45 to 1·33)
Abdominal hernias[e]	39 / 4036 (1·0%)	43 / 4087 (1·0%)	0·92 (0·52 to 1·62)
**Denominator –women eligible to be assessed**	**4783**	**4816**	
Woman's death	24 / 4773 (0·5%)	25 / 4805 (0·5%)	0·96 (0·50 to 2·00)
Reproductive status			
**Denominator - Women with no subsequent pregnancy**	**2350**	**2382**	
Voluntary infertility	2238 / 2350 (95·2%)	2262 / 2382 (95·0%)	1·00 (0·99 to 1·02)
**Denominator – women assessed**	**4036**	**4087**	
Voluntary infertility	2238 / 4036 (55·5%)	2622 / 4087 (64·2%)	1·00 (0·95 to 1·05)
Subsequent pregnancies			
**Denominator – women assessed**	**4036**	**4087**	
Any subsequent pregnancy	1686 / 4036 (42·0%)	1705 / 4087 (42·0%)	1·00 (0·94 to 1·07)
**Denominator – women with a subsequent pregnancy**	**1686**	**1705**	
Interpregnancy interval (months)[f]<br>Mean [sd]<br>Median {IQR}	 28·7 [11·6]<br>27·8 {19.9 to 35·9}	 28·3 [12·1]<br>27·0 {18·9 to 36·3}	 HR<br>1·00 (0·91 to 1·09)
**Denominator - women with a subsequent viable pregnancy**	**1411**	**1438**	
Bladder or bowel damage[j]	1 / 1208 (0·08%)	1 / 1209 (0·08%)	1·00 (0·02 to 38·17)
**Denominator - women assessed**	**4036**	**4087**	
Bladder or bowel damage[j]	1 / 3816 (0·03%)	1 / 3828(0·03%)	1·00 (0·03 to 38·30)

[a] Following the CORONIS birth and before any subsequent pregnancy, any new onset or worsening.
[b] Urinary symptoms of poor stream and/or frequency which did not respond to antibiotics.
[c] Not related to pregnancy.
[d] In those women who have had surgery excluding diagnostic laparoscopy and diagnostic laparotomy.
[e] Following the CORONIS birth any new onset.
[f] From the CORONIS birth to the end of the subsequent pregnancy (regardless of loss or birth·)
[g] Of the first viable pregnancy (gestational age > 24 or >28 weeks depending on country specific definition.
[h] For the birth following the CORONIS birth.
[i] Includes: uterine rupture, uterine scar dehiscence, placenta praevia, morbidly adherent placenta, abruption, postpartum haemorrhage requiring transfusion, severe infection within 6 weeks, postpartum hysterectomy up to 6 weeks postpartum, manual removal of placenta.
[j] At the time of subsequent Caesarean section.

Table S13 Uterine repair suture

Outcome	Catgut	PG-910	Adjusted risk ratio, mean difference (MD) or hazards ratio (HR) (99% CI)
Women's health and mortality			
**Denominator – women assessed**	**3974**	**3972**	
Pelvic pain[a]	202 / 3974(5·1%)	218 / 3972 (5·5%)	0·93 (0·73 to 1·18)
Dysmenorrhoea[a]	191 / 3974 (4·8%)	207 / 3972 (5·2%)	0·92 (0·72 to 1·19)
Deep dyspareunia[a]	116 / 3974 (2·9%)	133 / 3972 (3·4%)	0·87 (0·63 to 1·20)
Urinary symptoms[a·b]	43 / 3974 (1·1%)	45 / 3972 (1·1%)	0·95 (0·55 to 1·65)
Diagnostic laparoscopy/laparotomy[c]	2 / 3974 (0·05%)	2 / 3972 (0·05%)	1·00 (0·76 to 13·15)
Hysterectomy or tubal/ovarian surgery[c]	11 / 3974 (0·3%)	10 / 3972 (0·3%)	1·09 (0·37 to 3·37)
Bladder or bowel damage[cd]	1 / 72 (1·4%)	0 / 74 (0·0%)	-
Bowel obstruction[e]	2 /3974 (0·05%)	0 / 3972 (0·00%)	-
Reproductive status			
**Denominator – women assessed**	**3974**	**3972**	
Involuntary infertility	162 / 3974 (4·1%)	182 / 3972 (4·6%)	0·88 (0·68 to 1·16)
Use of fertility treatments	20 / 3972 (0·5%)	16 / 3971 (0·4%)	1·24 (0·53 to 2·94)
Subsequent pregnancies			
**Denominator – women assessed**	**3974**	**3972**	
Any subsequent pregnancy	1861 / 3974 (46·8%)	1842 / 3972 (46·4%)	1·00 (0·95 to 1·07)
**Denominator – women with a subsequent pregnancy**	**1861**	**1842**	
Interpregnancy interval (months)[f]			
Mean [sd]	28·9 [11·8]	29·6 [11·5]	HR
Median {IQR}	27·5 {20·2 to 36·4}	28·7 {20·7 to 36·9}	1·04 (0·95 to 1·13)
Miscarriage	236 / 1844 (13·0%)	210 / 1833 (11·5%)	1·11 (0·89 to 1·40)
Ectopic pregnancy	4 / 1844 (0·2%)	4 / 1833 (0·2%)	1·00 (0·16 to 6·15)
**Denominator - women with a subsequent viable pregnancy**	**1661**	**1648**	
Gestation at delivery (completed wks)[g]			MD
Mean [sd]	38·1 [1·4]	38·2 [1·3]	-0·07 (-0·19 to 0·06)
Median {IQR}	38·6 {38·0 to 38·6}	38·6 {38·0 to 38·6}	
Bladder or bowel damage[j]	1 / 1449 (0·07%)	2 / 1429 (0·14%)	0·49 (0·02 to 11.54)
**Denominator - women assessed**	**3974**	**3972**	
Bladder or bowel damage[j]	1 / 3745 (0·03%)	2 / 3744 (0·05%)	0·50 (0·02 to 11·71)

[a] Following the CORONIS birth and before any subsequent pregnancy, any new onset or worsening.
[b] Urinary symptoms of poor stream and/or frequency which did not respond to antibiotics.
[c] Not related to pregnancy.
[d] In those women who have had surgery excluding diagnostic laparoscopy and diagnostic laparotomy.
[e] Following the CORONIS birth any new onset.
[f] From the CORONIS birth to the end of the subsequent pregnancy (regardless of loss or birth·)
[g] Of the first viable pregnancy (gestational age > 24 or >28 weeks depending on country specific definition.
[h] For the birth following the CORONIS birth.
[i] Includes: uterine rupture, uterine scar dehiscence, placenta praevia, morbidly adherent placenta, abruption, postpartum haemorrhage requiring transfusion, severe infection within 6 weeks, postpartum hysterectomy up to 6 weeks postpartum, manual removal of placenta.
[j] At the time of subsequent Caesarean section.

Table S14 All outcomes by sub-continent and overall

	South America	East and West Africa	South Asia	Totals
Women's health and mortality				
**Denominator – women assessed**	**2405**	**3785**	**6963**	**13153**
Pelvic pain[a]	138 / 2405 (5·7%)	180 / 3785 (4·8%)	346 / 6963 (5·0%)	664 / 13153 (5·0%)
Dysmenorrhoea[a]	252 / 2405 (10·5%)	191 / 3785 (5·0%)	271 / 6963 (3·9%)	714 / 13153 (5·4%)
Deep dyspareunia[a]	150 / 2405 (6·2%)	128 / 3785 (3·4%)	152 / 6963 (2·2%)	430 / 13153 (3·3%)
Urinary symptoms[ab]	43 / 2405 (1·8%)	40 / 3785 (1·1%)	54 / 6963 (0·8%)	137 / 13153 (1·0%)
Diagnostic laparoscopy/laparotomy[b]	5 / 2405 (0·2%)	2 / 3783 (0·1%)	0 / 6963 (0·0%)	7 / 13151 (0·1%)
Hysterectomy or tubal/ovarian surgery[b]	11 / 2405 (0·5%)	8 / 3785 (0·2%)	11 / 6963 (0·2%)	30 / 13153 (0·2%)
Bladder or bowel damage[bc]	0 / 115 (0·0%)	1 / 36 (2·8%)	0 / 113 (0·0%)	1 / 264 (0·4%)
Abdominal hernias[d]	37 / 2405 (1·5%)	18 / 3785 (0·5%)	47 / 6963 (0·7%)	102 / 13153 (0·8%)
Bowel obstruction[d]	1 / 2405 (0·04%)	2 / 3785 (0·05%)	2 / 6963 (0·03%)	5 / 13153 (0·04%)
**Denominator – women eligible to be assessed**	**2782**	**4504**	**8347**	**15633**
Woman's death	9 / 2782 (0·3%)	32 / 4504 (0·7%)	48 / 8347 (0·6%)	89 / 15633 (0·6%)
Reproductive status				
**Denominator - women with no subsequent pregnancy**	**1800**	**2028**	**3510**	**7338**
Voluntary infertility	1773 / 1800 (98·5%)	1773 / 2028 (87·4%)	3276 / 3510 (93·3%)	6822 / 7338 (93·0%)
**Denominator – women assessed**	**2405**	**3785**	**6963**	**13153**
Involuntary infertility	23 / 2405 (1·0%)	201 / 3785 (5·3%)	191 / 6963 (2·7%)	415 / 13153 (3·2%)
Use of fertility treatments	0 / 2403 (0·0%)	19 / 3784 (0·5%)	27 / 6963 (0·4%)	46 / 13150 (0·3%)
Subsequent pregnancies				
**Denominator – women assessed**	**2405**	**3785**	**6963**	**13153**
Any subsequent pregnancy	605 / 2405 (25·2%)	1757 / 3785 (46·4%)	3453 / 6963 (49·6%)	5815 / 13153 (44·2%)
**Denominator – women with a subsequent pregnancy**	**605**	**1757**	**3453**	**5815**
Interpregnancy interval (months)[e]				
Mean [sd]	31·9 [12·9]	31·6 [11·9]	27·7 [11·5]	29·3 [11·9]
Median {IQR}	31·5 {21·6 to 39·1}	30·4 {22·6 to 39·3}	26·7 {19·0 to 35·0}	28·3 {20·3 to 36·8}
Miscarriage (excludes women pregnant at the time of assessment)	52 / 584 (8·9%)	201 / 1752 (11·5%)	453 / 3415 (13·3%)	706 / 5751 (12·3%)
Ectopic pregnancy (excludes women pregnant at the time of assessment)	4 / 584 (0·7%)	4 / 1752 (0·2%)	8 / 3415 (0·2%)	16 / 5751 (0·3%)

Table S14 continued	South America	East and West Africa	South Asia	Totals
**Denominator - women with a subsequent viable pregnancy[f]**	**536**	**1604**	**2852**	**4992**
Gestation at delivery (completed wks)				
Mean [sd]	38·4 [1·9]	38·4 [1·3]	38·0 [1·4]	38·2 [1·5]
Median {IQR}	38·6 {38·0 to 39·0}	38·6 {38·0 to 38·6}	38·0 {38·0 to 38·6}	38·6 {38·0 to 38·6}
Stillbirth[e]	3 / 541 (0·6%)	45 / 1630 (2·8%)	44 / 2879 (1·5%)	92 / 5050 (1·8%)
Neonatal death[e]	6 / 538 (1·1%)	27 / 1582 (1·7%)	54 / 2835 (1·9%)	87 / 4955 (1·8%)
Mode of delivery[e]				
Non-instrumental vaginal	65 / 541 (12·0%)	265 / 1629 (16·3%)	428 / 2879 (14·9%)	758 / 5049 (15·0%)
Instrumental vaginal	1 / 541 (0·2%)	3 / 1629 (0·2%)	53 / 2879 (1·8%)	57 / 5049 (1·1%)
Pre-labour caesarean section	424 / 541 (78·4%)	1089 / 1629 (66·9%)	1815 / 2879 (63·0%)	3328 / 5049 (65·9%)
In labour caesarean section	51 / 541 (9·4%)	272 / 1629 (16·7%)	583 / 2879 (20·3%)	906 / 5049 (17·9%)
All caesarean sections[e]	475 / 541 (87·8%)	1361 / 1629 (83·5%)	2398 / 2879 (83·3%)	4234 / 5049 (83·9%)
**Denominator - women with a subsequent viable pregnancy[f]**	**536**	**1604**	**2852**	**4992**
Other pregnancy complications – composite[eg]	29 / 535 (5·4%)	44 / 1601 (2·7%)	56 / 2851 (2·0%)	129 / 4987 (2·6%)
Uterine rupture [e]	0 / 536 (0·00%)	1 / 1602 (0·06%)	4 / 2852 (0·14%)	5 / 4990 (0·10%)
Uterine scar dehiscence[e]	2 / 536 (0·4%)	3 / 1601 (0·2%)	5 / 2852 (0·2%)	10 / 4989 (0·2%)
Placenta praevia[e]	3 / 536 (0·6%)	9 / 1601 (0·6%)	9 / 2852 (0·3%)	21 / 4989 (0·4%)
Morbidly adherent placenta[e]	1 / 536 (0·19%)	1 / 1601 (0·06%)	5 / 2852 (0·18%)	7 / 4989 (0·14%)
Abruption[e]	1 / 536 (0·2%)	5 / 1602 (0·3%)	8 / 2852 (0·3%)	14 / 4990 (0·3%)
Postpartum haemorrhage requiring transfusion of >1 unit of whole blood or packed cells[e]	3 / 536 (0·6%)	9 / 1602 (0·6%)	10 / 2852 (0·4%)	22 / 4990 (0·4%)
Severe infection within 6 weeks postpartum[e]	14 / 535 (2·6%)	10 / 1602 (0·6%)	25 / 2851 (0·9%)	49 / 4988 (1·0%)
Hysterectomy up to 6 weeks postpartum[e]	1 / 536 (0·19%)	2 / 1603 (0·12%)	2 / 2852 (0·07%)	5 / 4991 (0·10%)
Manual removal of placenta[e]	6 / 536 (1·1%)	9 / 1603 (0·6%)	3 / 2852 (0·1%)	18 / 4991 (0·4%)
Bladder or bowel damage[h]	0 / 471 (0·0%)	1 / 1339 (0·07%)	2 / 2375 (0·08%)	3 / 4185 (0·07%)
CORONIS child(ren)				
**Denominator – live born children of women eligible to be assessed**	**2866**	**4658**	**8574**	**16098**
Known deaths or serious morbidity of CORONIS child(ren)	113 / 2866 (3·9%)	288 / 4658 (6·2%)	351 / 8574 (4·1%)	752 / 16098 (4·7%)

[a] Following the CORONIS birth and before any subsequent pregnancy, any new onset or worsening.

[b] Not related to pregnancy.

[c] Excluding diagnostic laparoscopy and diagnostic laparotomy.

[d] Following the CORONIS birth, any new onset.

[e] For the birth following the CORONIS birth

[f] Viability could not be assessed if gestational age was not recorded

[g] Includes: uterine rupture, uterine scar dehiscence, placenta praevia, morbidly adherent placenta, abruption, postpartum haemorrhage requiring transfusion,

severe infection within 6 weeks postpartum, hysterectomy up to 6 weeks postpartum, manual removal of placenta.

[h] at the time of subsequent Caesarean section.

Missing data are <1% unless otherwise stated.

Table S15 All outcomes by region and overall

	Argentina	Chile	Ghana	India – Delhi	India – Vellore	Kenya	Pakistan	Sudan	Totals
Women's health and mortality									
**Denominator – women assessed**	**1271**	**1134**	**1041**	**2862**	**2063**	**1446**	**2038**	**1298**	**13153**
Pelvic pain[a]	84 / 1271 (6.6%)	54 / 1134 (4.8%)	41 / 1041 (3.9%)	84 / 2862 (2.9%)	35 / 2063 (1.7%)	54 / 1446 (3.7%)	227 / 2038 (11.1%)	85 / 1298 (6.5%)	664 / 13153 (5.0%)
Dysmenorrhoea[a]	159 / 1271 (12.5%)	93 / 1134 (8.2%)	43 / 1041 (4.1%)	71 / 2862 (2.5%)	42 / 2063 (2.0%)	52 / 1446 (3.6%)	158 / 2038 (7.8%)	96 / 1298 (7.4%)	714 / 13153 (5.4%)
Deep dyspareunia[a]	106 / 1271 (8.3%)	44 / 1134 (3.9%)	37 / 1041 (3.6%)	29 / 2862 (1.0%)	20 / 2063 (1.0%)	40 / 1446 (2.8%)	103 / 2038 (5.1%)	51 / 1298 (3.9%)	430 / 13153 (3.3%)
Urinary symptoms[a.b]	25 / 1271 (2.0%)	18 / 1134 (1.6%)	18 / 1041 (1.7%)	7 / 2862 (0.2%)	9 / 2063 (0.4%)	13 / 1446 (0.9%)	38 / 2038 (1.9%)	9 / 1298 (0.7%)	137 / 13153 (1.0%)
Diagnostic laparoscopy/laparotomy[b]	2 / 1271 (0.2%)	3 / 1134 (0.3%)	0 / 1039 (0.0%)	0 / 2862 (0.0%)	0 / 2063 (0.0%)	1 / 1446 (0.1%)	0 / 2038 (0.0%)	1 / 1298 (0.1%)	7 / 13151 (0.1%)
Hysterectomy or tubal/ovarian surgery[b]	5 / 1271 (0.4%)	6 / 1134 (0.5%)	2 / 1041 (0.2%)	2 / 2862 (0.1%)	6 / 2063 (0.3%)	6 / 1446 (0.4%)	3 / 2038 (0.1%)	0 / 1298 (0.0%)	30 / 13153 (0.2%)
Bladder or bowel damage[b,c]	0 / 52 (0.0%)	0 / 63 (0.0%)	0 / 5 (0.0%)	0 / 35 (0.0%)	0 / 48 (0.0%)	1 / 15 (6.7%)	0 / 30 (0.0%)	0 / 16 (0.0%)	1 / 264 (0.4%)
Abdominal hernias[d]	21 / 1271 (1.7%)	16 / 1134 (1.4%)	2 / 1041 (0.2%)	8 / 2862 (0.3%)	33 / 2063 (1.6%)	2 / 1446 (0.1%)	6 / 2038 (0.3%)	14 / 1298 (1.1%)	102 / 13153 (0.8%)
Bowel obstruction[d]	1 / 1271 (0.08%)	0 / 1134 (0.0%)	0 / 1041 (0.0%)	2 / 2862 (0.07%)	0 / 2063 (0.0%)	2 / 1446 (0.14%)	0 / 2038 (0.0%)	0 / 1298 (0.0%)	5 / 13153 (0.04%)
**Denominator – women eligible to be assessed**	**1611**	**1171**	**1267**	**3753**	**2086**	**1644**	**2508**	**1593**	**15633**
Woman's death	6 / 1611 (0.4%)	3 / 1171 (0.3%)	10 / 1267 (0.8%)	27 / 3753 (0.7%)	9 / 2086 (0.4%)	16 / 1644 (1.0%)	12 / 2508 (0.5%)	6 / 1593 (0.4%)	89 / 15633 (0.6%)
Reproductive status									
**Denominator - Women with no subsequent pregnancy**	**873**	**927**	**498**	**1535**	**1277**	**965**	**698**	**565**	**7338**
Voluntary infertility	857 / 873 (98.2%)	916 / 927 (98.8%)	422 / 498 (84.7%)	1483 / 1535 (96.6%)	1180 / 1277 (92.4%)	933 / 965 (96.7%)	613 / 698 (87.8%)	418 / 565 (74.0%)	6822 / 7338 (93.0%)

Table S15 continued

**Denominator – women assessed**	**1271**	**1134**	**1041**	**2862**	**2063**	**1446**	**2038**	**1298**	**13153**
Involuntary infertility	13 / 1271 (1.0%)	10 / 1134 (0.9%)	63 / 1041 (6.1%)	39 / 2862 (1.4%)	86 / 2063 (4.2%)	27 / 1446 (1.9%)	66 / 2038 (3.2%)	111 / 1298 (8.6%)	415 / 13153 (3.2%)
Use of fertility treatments	0 / 1271 (0.0%)	0 / 1132 (0.0%)	5 / 1040 (0.5%)	7 / 2862 (0.2%)	9 / 2063 (0.4%)	3 / 1446 (0.2%)	11 / 2038 (0.5%)	11 / 1298 (0.9%)	46 / 13150 (0.3%)
Subsequent pregnancies									
**Denominator – women assessed**	**1271**	**1134**	**1041**	**2862**	**2063**	**1446**	**2038**	**1298**	**13153**
Any subsequent pregnancy	398 / 1271 (31.3%)	207 / 1134 (18.3%)	543 / 1041 (52.2%)	1327 / 2862 (46.4%)	786 / 2063 (38.1%)	481 / 1446 (33.3%)	1340 / 2038 (65.8%)	733 / 1298 (56.5%)	5815 / 13153 (44.2%)
**Denominator – women with a subsequent pregnancy**	**398**	**207**	**543**	**1327**	**786**	**481**	**1340**	**733**	**5815**
Interpregnancy interval (months)[e]									
Mean [sd]	33.8 [14.1]	28.2 [9.1]	32.9 [12.9]	29.3 [12.4]	27.8 [11.6]	32.1 [12.2]	26.1 [10.3]	30.3 [10.8]	29.3 [11.9]
Median {IQR}	33.1 {22.1 to 42.8}	28.8{21.0 to 35.5}	31.3 {22.7 to 41.5}	28.7 {20.1 to 37.0}	26.4 {19.1 to 35.7}	31.1 {22.9 to 39.5}	24.9 {18.3 to 32.8}	29.4 {22.2 to 37.0}	28.3 {20.3 to 36.8}
Miscarriage[f]	28 / 381 (7.3%)	24 / 203 (11.8%)	66 / 541 (12.2%)	175 / 1312 (13.3%)	73 / 782 (9.3%)	50 / 480 (10.4%)	205 / 1321 (15.5%)	85 / 731 (11.6%)	706 / 5751 (12.3%)
Ectopic pregnancy[f]	1 / 381 (0.3%)	3 / 203 (1.5%)	1 / 541 (0.2%)	3 / 1312 (0.2%)	2 / 782 (0.3%)	1 / 480 (0.2%)	3 / 1321 (0.2%)	2 / 731 (0.3%)	16 / 5751 (0.3%)
**Denominator - women with a subsequent viable pregnancy[g]**	**359**	**177**	**492**	**986**	**662**	**438**	**1204**	**674**	**4992**
Gestation at delivery (completed wks)									
Mean [sd]	38.5 [2.0]	38.2 [1.7]	38.3 [1.2]	38.3 [1.6]	38.2 [1.6]	38.6 [1.9]	37.8 [1.2]	38.3 [0.9]	38.2 [1.5]
Median {IQR}	38.6 {38.0 to 39.0}	38.0 {38.0 to 39.0}	38.6 {38.6 to 38.6}	38.6 {38.0 to 38.6}	38.0 {38.0 to 39.0}	38.6 {38.0 to 40.0}	38.0 {38.0 to 38.0}	38.6 {38.0 to 38.6]	38.6 {38.0 to 38.6}
**Denominator – babies of women with a subsequent viable pregnancy[g]**	**361**	**180**	**504**	**992**	**668**	**443**	**1219**	**683**	**5050**
Stillbirth[e]	2 / 361 (0.6%)	1 / 180 (0.6%)	20 / 504 (4.0%)	12 / 992 (1.2%)	9 / 668 (1.4%)	12 / 443 (2.7%)	23 / 1219 (1.9%)	13 / 683 (1.9%)	92 / 5050 (1.8%)
Neonatal death[e,h]	6 / 359 (1.7%)	0 / 179 (0.0%)	8 / 481 (1.7%)	17 / 980 (1.7%)	5 / 659 (0.8%)	8 / 431 (1.9%)	32 / 1196 (2.7%)	11 / 670 (1.6%)	87 / 4955 (1.8%)

Table S15 continued

	Argentina	Chile	Ghana	India – Delhi	India – Vellore	Kenya	Pakistan	Sudan	Totals
Mode of delivery[e]									
Non-instrumental vaginal	50 / 361 (13.9%)	15 / 180 (8.3%)	146 / 504 (29.0%)	292 / 992 (29.4%)	28 / 668 (4.2%)	56 / 442 (12.7%)	108 / 1219 (8.9%)	63 / 683 (9.2%)	758 / 5049 (15.0%)
Instrumental vaginal	0 / 361 (0.0%)	1 / 180 (0.6%)	3 / 504 (0.6%)	10 / 992 (1.0%)	43 / 668 (6.4%)	0 / 442 (0.0%)	0 / 1219 (0.0%)	0 / 683 (0.0%)	57 / 5049 (1.1%)
Pre-labour caesarean section	276 / 361 (76.5%)	148 / 180 (82.2%)	270 / 504 (53.6%)	287 / 992 (28.9%)	433 / 668 (64.8%)	262 / 442 (59.3%)	1095 / 1219 (89.8%)	557 / 683 (81.6%)	3328 / 5049 (65.9%)
In labour caesarean section	35 / 361 (9.7%)	16 / 180 (8.9%)	85 / 504 (16.9%)	403 / 992 (40.6%)	164 / 668 (24.6%)	124 / 442 (28.1%)	16 / 1219 (1.3%)	63 / 683 (9.2%)	906 / 5049 (17.9%)
All caesarean sections[e]	311 / 361 (86.1%)	164 / 180 (91.1%)	355 / 504 (70.4%)	690 / 992 (69.6%)	597 / 668 (89.4%)	386 / 442 (87.3%)	1111 / 1219 (91.1%)	620 / 683 (90.8%)	4234 / 5049 (83.9%)
**Denominator - women with a subsequent viable pregnancy[g]**	**359**	**177**	**492**	**986**	**662**	**438**	**1204**	**674**	**4992**
Other pregnancy complications – composite[e,i]	23 / 358 (6.4%)	6 / 177 (3.4%)	10 / 490 (2.0%)	22 / 985 (2.2%)	16 / 662 (2.4%)	13 / 437 (3.0%)	18 / 1204 (1.5%)	21 / 674 (3.1%)	129 / 4987 (2.6%)
Uterine rupture[e]	0 / 359 (0.0%)	0 / 177 (0.0%)	1 / 491 (0.2%)	1 / 986 (0.1%)	1 / 662 (0.2%)	0 / 437 (0.0%)	2 / 1204 (0.2%)	0 / 674 (0.0%)	5 / 4990 (0.1%)
Uterine scar dehiscence[e]	2 / 359 (0.6%)	0 / 177 (0.0%)	0 / 490 (0.0%)	3 / 986 (0.3%)	2 / 662 (0.3%)	0 / 437 (0.0%)	0 / 1204 (0.0%)	3 / 674 (0.4%)	10 / 4989 (0.2%)
Placenta praevia[e]	3 / 359 (0.8%)	0 / 177 (0.0%)	3 / 490 (0.6%)	2 / 986 (0.2%)	3 / 662 (0.5%)	3 / 437 (0.7%)	4 / 1204 (0.3%)	3 / 674 (0.4%)	21 / 4989 (0.4%)
Morbidly adherent placenta[e]	1 / 359 (0.3%)	0 / 177 (0.0%)	0 / 490 (0.0%)	1 / 986 (0.1%)	3 / 662 (0.5%)	0 / 437 (0.0%)	1 / 1204 (0.1%)	1 / 674 (0.1%)	7 / 4989 (0.1%)
Abruption[e]	0 / 359 (0.0%)	1 / 177 (0.6%)	2 / 491 (0.4%)	5 / 986 (0.5%)	2 / 662 (0.3%)	1 / 437 (0.2%)	1 / 1204 (0.1%)	2 / 674 (0.3%)	14 / 4990 (0.3%)
Postpartum haemorrhage requiring transfusion of >1 unit of whole blood or packed cells[e]	1 / 359 (0.3%)	2 / 177 (1.1%)	2 / 491 (0.4%)	4 / 986 (0.4%)	3 / 662 (0.5%)	1 / 437 (0.2%)	3 / 1204 (0.2%)	6 / 674 (0.9%)	22 / 4990 (0.4%)

	Argentina	Chile	Ghana	India – Delhi	India – Vellore	Kenya	Pakistan	Sudan	Totals
Severe infection within 6 weeks postpartum[e]	11 / 358 (3.1%)	3 / 177 (1.7%)	2 / 491 (0.4%)	9 / 985 (0.9%)	7 / 662 (1.1%)	1 / 437 (0.2%)	9 / 1204 (0.7%)	7 / 674 (1.0%)	49 / 4988 (1.0%)
Hysterectomy up to 6 weeks postpartum[e]	1 / 359 (0.3%)	0 / 177 (0.0%)	0 / 491 (0.0%)	1 / 986 (0.1%)	1 / 662 (0.2%)	1 / 438 (0.2%)	0 / 1204 (0.0%)	1 / 674 (0.1%)	5 / 4991 (0.1%)

Table S15 continued

Manual removal of placenta[e]	6 / 359 (1.7%)	0 / 177 (0.0%)	1 / 491 (0.2%)	0 / 986 (0.0%)	1 / 662 (0.2%)	7 / 438 (1.6%)	2 / 1204 (0.2%)	1 / 674 (0.1%)	18 / 4991 (0.4%)
Bladder or bowel damage[j]	0 / 310 (0.0%)	0 / 161 (0.0%)	1 / 345 (0.3%)	0 / 687 (0.0%)	2 / 592 (0.3%)	0 / 382 (0.0%)	0 / 1096 (0.0%)	0 / 612 (0.0%)	3 / 4185 (0.1%)
CORONIS child(ren) **Denominator – live born children of women eligible to be assessed**	**1656**	**1210**	**1307**	**3860**	**2178**	**1689**	**2536**	**1662**	**16098**
Known deaths or serious morbidity of CORONIS child(ren)	82 / 1656 (5.0%)	31 / 1210 (2.6%)	104 / 1307 (8.0%)	201 / 3860 (5.2%)	54 / 2178 (2.5%)	126 / 1689 (7.5%)	96 / 2536 (3.8%)	58 / 1662 (3.5%)	752 / 16098 (4.7%)

[a] Following the CORONIS birth and before any subsequent pregnancy, any new onset or worsening.

[b] Not related to pregnancy.

[c] Excluding diagnostic laparoscopy and diagnostic laparotomy.

[d] Following the CORONIS birth, any new onset.

[e] For the birth following the CORONIS birth

[f] Excludes women pregnant at time of assessment.

[g] Viability could not be assessed if gestational age was not recorded

h Excludes stillbirths.

[i] Includes: uterine rupture, uterine scar dehiscence, placenta praevia, morbidly adherent placenta, abruption, postpartum haemorrhage requiring transfusion, severe infection within 6 weeks postpartum, hysterectomy up to 6 weeks postpartum, manual removal of placenta.

[j] At the time of subsequent Caesarean section.

Missing data are <1% unless otherwise stated.
